# SweC and SweD are essential co-factors of the FtsEX-CwlO cell wall hydrolase complex in *Bacillus subtilis*

**DOI:** 10.1371/journal.pgen.1008296

**Published:** 2019-08-22

**Authors:** Yannick R. Brunet, Xindan Wang, David Z. Rudner

**Affiliations:** Department of Microbiology, Harvard Medical School, Boston, MA, United States of America; Indiana University, UNITED STATES

## Abstract

The peptidoglycan (PG) sacculus is composed of long glycan strands cross-linked together by short peptides forming a covalently closed meshwork that protects the bacterial cell from osmotic lysis and specifies its shape. PG hydrolases play essential roles in remodeling this three-dimensional network during growth and division but how these autolytic enzymes are regulated remains poorly understood. The FtsEX ABC transporter-like complex has emerged as a broadly conserved regulatory module in controlling cell wall hydrolases in diverse bacterial species. In most characterized examples, this complex regulates distinct PG hydrolases involved in cell division and is intimately associated with the cytokinetic machinery called the divisome. However, in the gram-positive bacterium *Bacillus subtilis* the FtsEX complex is required for cell wall elongation where it regulates the PG hydrolase CwlO that acts along the lateral cell wall. To investigate whether additional factors are required for FtsEX function outside the divisome, we performed a synthetic lethal screen taking advantage of the conditional essentiality of CwlO. This screen identified two uncharacterized factors (SweD and SweC) that are required for CwlO activity. We demonstrate that these proteins reside in a membrane complex with FtsX and that amino acid substitutions in residues adjacent to the ATPase domain of FtsE partially bypass the requirement for them. Collectively our data indicate that SweD and SweC function as essential co-factors of FtsEX in controlling CwlO during cell wall elongation. We propose that factors analogous to SweDC function to support FtsEX activity outside the divisome in other bacteria.

## Author summary

Bacterial growth and division require the synthesis and remodeling of the cell wall exoskeleton. To prevent lethal breaches in this protective layer, peptidoglycan (PG) hydrolases that remodel the cell wall must be carefully regulated but the mechanisms underlying this control remain poorly understood. The noncanonical ABC transporter FtsEX has emerged as a broadly conserved regulator of PG hydrolases. In most characterized examples, FtsEX is integrated into the division machinery where it controls cell wall cleavage during cytokinesis. By contrast, in *Bacillus subtilis* the FtsEX complex functions in cell wall elongation. Here, we report the identification of two previously uncharacterized proteins (SweD and SweC) that function as essential co-factors of FtsEX in controlling PG hydrolase activity along the lateral cell wall. Homologs of SweD and SweC are found in a subset of firmicutes. We propose that these and analogous factors enable FtsEX to function outside the divisome to control cell wall elongation hydrolases.

## Introduction

Most bacteria are encased within a cell wall exoskeleton composed of the heteropolymer peptidoglycan (PG). This macromolecule is assembled from long glycan strands cross-linked together by attached peptides, generating a continuous three-dimensional meshwork that encapsulates the cytoplasmic membrane, specifies cell shape, and protects the cell from its internal turgor pressure [1, 2]. Bacterial growth and division are intimately linked to hydrolysis of this covalently closed exoskeleton. To enlarge this meshwork during growth, bonds connecting the glycan strands must be broken to allow expansion of the meshwork and/or to incorporate new strands between the existing ones [3–5]. Similarly, during cell division, bonds must be broken in the nascent septal PG to allow invagination of the outer membrane in gram-negative bacteria and to promote cell separation in gram-positive bacteria. PG hydrolases play a central role in these processes, but their activities must be tightly regulated to prevent excessive degradation of the cell wall and the generation of lethal breaches in this protective layer. Many of the enzymes responsible for cell growth and division have been identified in a growing number of bacteria [6–20]. However, the mechanisms by which they are regulated remain incompletely understood. A deeper understanding of these regulatory systems has the potential to reveal new ways to subvert PG biogenesis for therapeutic intervention [4, 21].

Progress in our understanding of the control of PG hydrolysis comes from studies of the broadly conserved FtsEX complex. This substrate-less ABC transporter has been found to regulate distinct PG hydrolases in diverse bacterial species including *E*. *coli*, *Streptococcus pneumoniae*, *B*. *subtilis*, *Bacillus anthracis*, *Mycobacterium tuberculosis*, and *Caulobacter crescentus* [21–27]. FtsEX is a member of the Type VII ABC transporter superfamily that is thought to function in mechanotransmission rather than as transporters [28]. FtsE is the ATPase and FtsX is the transmembrane domain subunit of the complex. The large extracellular loops of FtsX interact with species-specific PG hydrolases that contain regulatory coiled-coil domains or, in the case of *E*. *coli*, an activator of PG hydrolases with a regulatory coiled-coil domain. Structural studies of the *S*. *pneumoniae* PG hydrolase PcsB that is controlled by FtsEX [26, 29] suggest that the coiled-coil domain resembles long molecular tweezers that hold the globular PG hydrolase domain in an inactive state [30]. Based on structural analysis of MacB, another member of the Type VII ABC transporter superfamily [28], FtsEX is thought to function by a mechanotransmission mechanism in which the ATPase cycle of FtsE controls conformational changes in the extracellular loop domains of FtsX triggering PG hydrolase activity perhaps by releasing the catalytic domain from the clutches of its regulatory coiled-coil domain.

In most bacteria in which this conserved regulatory module has been examined, FtsEX functions in the context of cell division. In the case of *E*. *coli*, FtsEX controls the activity of two PG amidases (AmiA and AmiB) via the coiled-coil domain-containing regulator EnvC [27, 31]. PG hydrolysis by these enzymes allows invagination of the outer membrane through the nascent septal PG layer during cytokinesis. FtsEX is intimately linked to divisome function. The complex is recruited to the cytokinetic ring at an early step in its assembly and recent work indicates that it interacts with the actin-like division protein FtsA to promote recruitment of downstream division factors [32, 33]. Furthermore, ATP hydrolysis by the FtsEX complex is not only required for cell wall hydrolysis by the two amidases it is also necessary for septal PG synthesis [32, 34] suggesting that FtsEX functions as a central coordinator of these two processes. FtsEX in *C*. *crescentus* has similarly been implicated in linking PG synthesis and its remodeling during cytokinesis [24]. In *S*. *pneumoniae*, FtsEX controls the PG hydrolase PcsB during cell division [26]. The mechanism by which FtsEX is recruited to the divisome is not known but, by analogy to *E*. *coli*, is thought to involve interactions with divisome components like FtsA or FtsZ [26]. The signal(s) that stimulate cycles of ATP hydrolysis in any of these systems are currently unknown but are likely intimately linked to the onset of PG synthesis during cytokinesis.

Interestingly, in *B*. *subtilis*, FtsEX is not involved in cell division but instead controls the PG hydrolase CwlO that plays a central role in cell wall elongation during growth [22, 25]. CwlO contains an amino-terminal coiled-coil domain followed by a NlpC/P60 D,L-endopeptidase domain that cleaves the bond between γ-D-glutamate and meso-diaminopimelic acid within the stem peptide [35]. CwlO and a second D,L-endopeptidase (LytE) [36] that is not regulated by FtsEX, are functionally redundant [6, 13]. Cells lacking either of these enzymes are viable but depletion of one in the absence of the other results in a lethal block to cell wall elongation. Consistent with the idea that CwlO is controlled by FtsEX, cells lacking either FtsE or FtsX or harboring point mutations in the putative ATPase domain of FtsE are blocked for cell wall elongation upon depletion of LytE [22, 25]. As in the case of the division-associated FtsEX complexes, the signals that stimulate FtsEX activity during growth are currently unknown. Here, we investigate whether other factors are required for FtsEX to function outside the multi-protein divisome complex. Taking advantage of the functional redundancy of CwlO and LytE we performed a synthetic lethal screen for mutants that require *lytE* for growth and identified two uncharacterized genes *yqzD* (*sweD*) and *yqzC* (*sweC*) that function in the same genetic pathway as *ftsEX* and *cwlO*. We demonstrate that SweD and SweC reside in a multimeric membrane complex with FtsX and function as essential co-factors of FtsEX in the control of CwlO activity. Orthologs of SweD and SweC are present in a subset of Bacilliaceae, Lactobacillales, and Listeriaceae suggesting these factors similarly enable FtsEX to control elongation hydrolases. Interestingly, Bernhardt and co-workers have identified two unrelated membrane proteins in *Corynebacterium glutamicum* that function as co-factors of the FtsEX-RipC cell wall hydrolase complex to promote cell separation in this organism. Thus, FtsEX complexes employ distinct co-factors to regulate PG hydrolases during cell division, separation, and elongation.

## Results

### Identification of *sweD* and *sweC*

**Fig 1:**
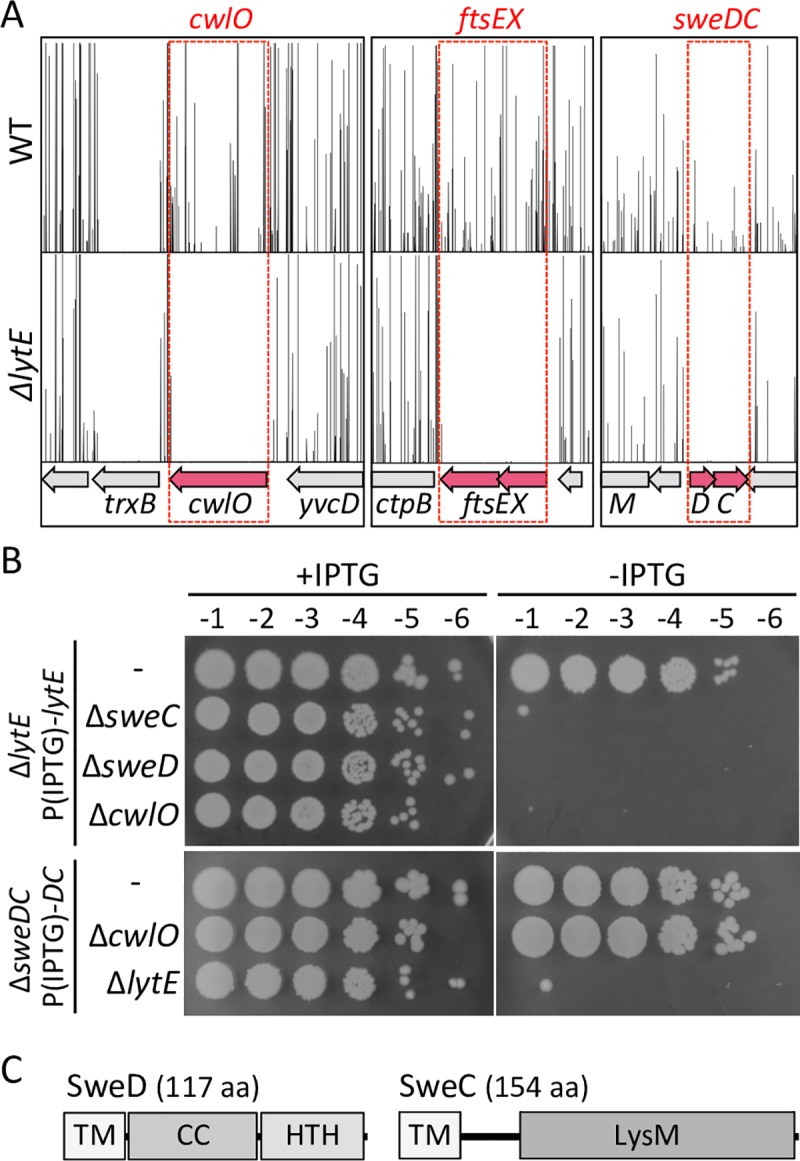
Transposon-sequencing identifies *sweD* and *sweC* as synthetic lethal with *lytE*. (**A**) Transposon insertion profiles from wild-type (WT) and Δ*lytE* libraries. Three regions of the genome are shown. The height of each line reflects the number of sequencing reads at this genomic position. Transposon insertions in *cwlO*, *ftsE*, *ftsX*, *sweD* (*yqzD*) and *sweC* (*yqzC*) are underrepresented in the Δ*lytE* library compared to WT. No insertions were mapped to the essential gene *trxB* in either library. (**B**) *sweD* and *sweC* mutants are synthetically lethal with Δ*lytE*. Spot dilutions of the indicated strains in the presence and absence of inducer. All strains were grown in the presence of IPTG (500 μM) to OD_600_ ~2.0. The cultures were washed twice without inducer, resuspended at an OD_600_ of 1.5, and 10-fold serially diluted. Five microliters of each dilution was spotted onto LB agar plates with and without IPTG. Representative plates from one of three biological replicates are shown. (**C**) Schematic representations of SweD and SweC. Transmembrane segments (TM); putative coiled-coil (CC); helix-turn-helix motif (HTH); and LysM homology domain are indicated.

To identify additional factors required for FtsEX-CwlO function, we took advantage of the synthetic lethal relationship between *cwlO* and *lytE*. Using transposon-sequencing (Tn-Seq) [37] we screened for genes that could tolerate transposon insertions in wild-type (LytE^+^) *B*. *subtilis* but not in cells lacking *lytE*. Such genes are predicted to function in a genetic pathway with *ftsEX* and *cwlO*. Mariner transposon libraries with >100,000 unique insertions were generated in wild-type *B*. *subtilis* PY79 and an isogenic Δ*lytE* variant. The two libraries were separately pooled and the transposon-chromosome junctions were mapped by massively parallel DNA sequencing. As anticipated and in validation of our screen, transposon insertions in *cwlO*, *ftsE*, and *ftsX* were readily detected in the wild-type library but were virtually undetectable in the library generated in the Δ*lytE* mutant (**Fig 1A**). In addition to these positive controls we identified two genes (*yqzD* and *yqzC*) of unknown function that were statistically (P<0.05 Mann-Whiney U test) underrepresented in the Δ*lytE* library compared to wild-type (**Fig 1A**). Based on the experiments presented below we have renamed these genes *sweD* and *sweC* for synthetic lethal with LytE.

The conditional essentiality of *sweD* and *sweC* was confirmed by depleting LytE in cells lacking either of these new factors. Fig 1B shows that depletion of LytE in *ΔsweD*, *ΔsweC*, or *ΔcwlO* mutants does not support colony formation. Similarly, cells lacking LytE that were depleted of SweD and SweC were inviable (**Fig 1B**). Consistent with the idea that *sweD* and *sweC* specifically function in a genetic pathway with *ftsEX* and *cwlO*, there was no plating defect in the Δ*cwlO* and Δ*ftsEX* mutants upon depletion of SweD and/or SweC (**Fig 1B and S1A Fig.**).

The *sweD* and *sweC* genes reside in an operon and are predicted to encode proteins of 13 kDa and 16 kDa, respectively (**Fig 1C**). Both proteins are predicted to contain N-terminal transmembrane (TM) segments. SweD contains a predicted coiled-coil (CC) domain followed by a C-terminal helix-turn-helix (HTH) motif (**Fig 1C**). The C-terminal region of SweC has remote homology to LysM domains that bind polysaccharides including chitin and peptidoglycan. Homologs of SweD and SweC are present in a subset of Bacilliaceae, Listeriaceae, and Lactobacillales family members (**S2 Fig**). PSI-BLAST also identified potential SweD and SweC homologs in the gram-negative bacterium *Helicobacter pylori* and a small group of unculturable bacteria that are similarly not firmicutes.

### Cytological characterization of *sweD* and *sweC* mutants

**Fig 2:**
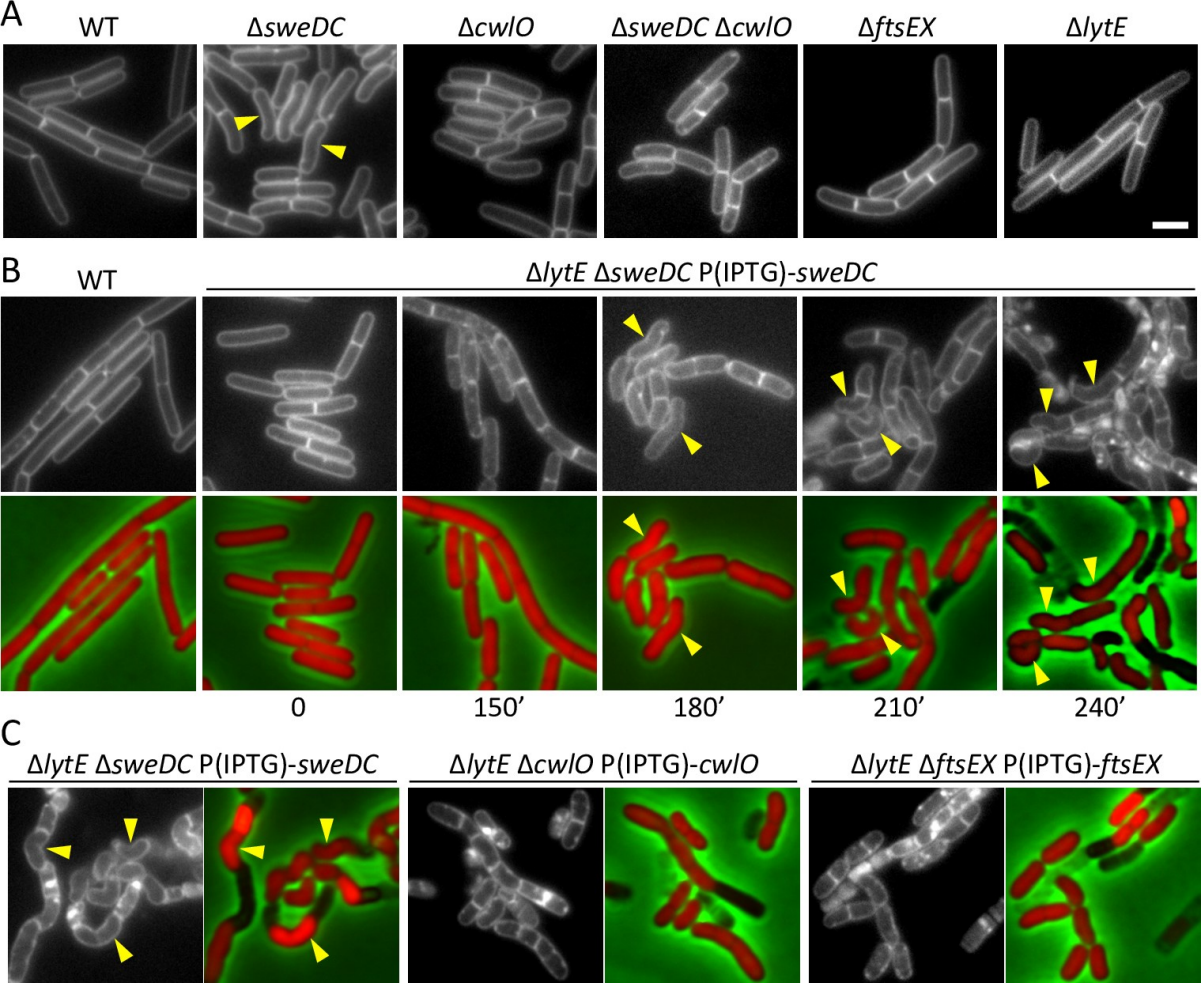
Δ*sweDC* mutants have morphological defects that resemble Δ*cwlO* and Δ*ftsEX*. **(A)** Representative images of wild-type (WT) (BDR2649), Δ*sweDC* (BYB370), Δ*cwlO* (BYB371), Δ*sweDC* Δ*cwlO* (BYB374), Δ*ftsEX* (BYB372), and Δ*lytE* (BYB373) are shown. Exponentially growing cells in CH medium were stained with the membrane dye TMA-DPH and examined by fluorescence microscopy. **(B)** Representative images of a Δ*lytE* strain during depletion of SweDC. The indicated strain (BYB362) harboring cytoplasmic mCherry was grown to exponential phase in CH medium in the presence of 500 μM IPTG, washed twice with medium lacking inducer, and used to inoculate CH medium at an OD_600_ of 0.05. The cells were examined by fluorescence microscopy at the indicated times. Membranes were visualized with TMA-DPH (top) and cytoplasmic mCherry and phase contrast (bottom). (**C**) Comparison of the terminal phenotypes of strains lacking LytE and depleted of SweDC (BYB362), CwlO (BYB279), or FtsEX (BYB439) The indicated strains were grown to exponential phase in the presence of IPTG (500 μM), washed twice with medium lacking inducer, back-diluted to an OD_600_ of 0.05 (BYB362, BYB439) or 0.1 (BYB279) in CH medium, and grown to mid-exponential phase in the absence of inducer. Cells were examined by fluorescence microscopy. Images of SweDC, CwlO, and FtsEX depletions are from 230, 60, and 150 min after removal of IPTG, respectively. The different times required to reach the terminal phenotype likely reflect the abundance and half-lives of the individual proteins. All strains contained cytoplasmic mCherry, and an overlay of mCherry and phase contrast is shown adjacent to TMA-DPH-stained membranes. The representative images in this figure are from one of three independent experiments. Curved cell morphologies are highlighted (yellow carets). Scale bar indicates 2 μm.

To investigate whether *sweD* and *sweC* are in the same genetic pathway as *cwlO* and *ftsEX*, we analyzed the cytological phenotypes of the mutants by fluorescence microscopy. Cells lacking CwlO or FtsEX (or both) are shorter and fatter than wild-type and often slightly curved or bent [6, 13, 25, 38]. Accordingly, we directly compared the morphologies of the Δ*sweDC* mutant to cells lacking CwlO or FtsEX. The cells were grown in defined rich (CH) medium and analyzed by fluorescence microscopy using the fluorescent membrane dye TMA-DPH. The majority of the Δ*sweDC* mutant cells were shorter and fatter than wild-type (**Fig 2A**). However, cells lacking *sweDC* appeared even more bent and curved than the Δ*cwlO* and Δ*ftsEX* mutants. Importantly, these phenotypes were lost in cells that lacked both SweDC *and* CwlO, suggesting that these morphological defects require CwlO (**Fig 2A**). Similar phenotypes were observed in the Δ*sweC* and Δ*sweD* single mutants (**S1B Fig**), but as shown below the two proteins depend upon each other for stability, making it difficult to assign specific functions to either factor. Finally, as reported previously [25], cells lacking the second elongation PG hydrolase LytE resembled wild-type (**Fig 2A**).

A Δ*lytE* mutant depleted of CwlO or FtsEX is impaired in elongation but remains capable of cell division, generating short fat cells that ultimately lyse. To further explore the role of SweDC function in cell elongation, we examined the morphological defects in Δ*lytE* cells upon depletion of SweDC (**Fig 2B**). Cells lacking LytE and harboring an IPTG-regulated *sweDC* allele were grown in the presence of inducer in rich medium. Cytoplasmic mCherry and the membrane dye TMA-DPH were visualized by fluorescence microscopy before and at 30-minute intervals after removal of IPTG. As can be seen in Fig 2B, 150 minutes after IPTG removal, cell length was slightly reduced and this reduction continued over the next 60 minutes followed by the onset of lysis. Quantitative image analysis revealed a ~30% reduction in cell length over the depletion time course, similar to what was observed in a Δ*lytE* mutant depleted of CwlO (**S3 Fig**) [25]. Consistent with the curved and bent phenotypes observed in the LytE+ Δ*sweDC* mutant (**Fig 2A**), depletion of SweD and SweC in the absence of LytE resulted in even more dramatic curved morphologies prior to lysis (**Fig 2B**). A comparison of the terminal phenotypes of cells depleted for SweDC, CwlO and FtsEX in a Δ*lytE* mutant (**Fig 2C**) revealed that all three depletions generate shorter cells prior to lysis, however the dramatic curved morphologies were only observed when SweDC was depleted. Importantly, this phenotype was suppressed when LytE was depleted in cells in which both CwlO and SweDC were absent (**S4 Fig**). Altogether, these results argue that *cwlO*, *ftsEX* and *sweDC* reside in the same genetic pathway, and support the idea that SweD and SweC are required for PG hydrolase activity of CwlO.

### SweD and SweC are type II integral membrane proteins

**Fig 3:**
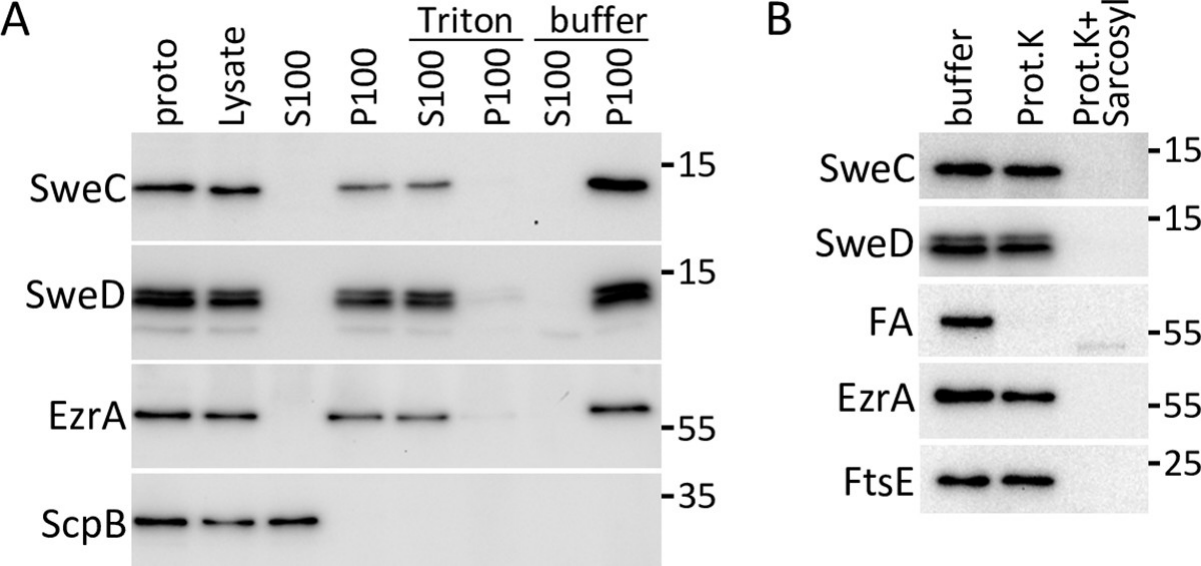
SweD and SweC are Type II membrane proteins. (**A**) Fractionation of SweD and SweC analyzed by immunoblot. Lysates from exponentially growing wild-type cells were subject to centrifugation to separate soluble (S100) and membrane-associated (P100) proteins. The membrane fraction was incubated with buffer or buffer supplemented with TritonX-100 and the solubilized proteins (S100) were separated from insoluble material (P100) by a second round of centrifugation. Equivalent amounts of each fraction including the input protoplasts (proto) were separated by SDS-PAGE and analyzed by immunoblot. The membrane protein EzrA and cytoplasmic protein ScpB served as membrane and cytoplasmic controls. (**B**) Protease accessibility analysis of SweD and SweC analyzed by immunoblot. Protoplasts of strain BDR776 were treated with buffer or buffer supplemented with Proteinase K in the absence or presence of sodium-lauroyl-sarcosinate (Sarcosyl). Reactions were resolved by SDS-PAGE and analyzed by immunoblot. SweD and SweC were inaccessible to cleavage by Proteinase K. Degradation of the extracellular domain of SpoIVFA (FA) served as a protease accessible control. The membrane protein EzrA and the cytoplasmic protein FtsE served as protease inaccessible controls. The immunoblots shown are from one of three independent experiments. Molecular weight markers (in kDa) are indicated.

To verify that SweD and SweC are integral membrane proteins and investigate their topologies, we raised antibodies against the soluble domains of both factors and analyzed the proteins by subcellular fractionation. Protoplasts were generated from exponentially growing wild-type cells and then lysed by addition of hypotonic buffer. The lysate was subjected to ultracentrifugation and the membrane fraction was homogenized with buffer in the presence and absence of the nonionic detergent TritonX-100 followed by a second round of centrifugation. As expected for integral membrane proteins, SweC and SweD fractionated with the membranes and could be solubilized with TritonX-100 (**Fig 3A**). The integral membrane protein EzrA [39] and cytoplasmic protein ScpB [40] served as positive and negative controls for this analysis.

Next, we investigated whether the soluble domains of SweC and SweD reside in the cytosol or on the extracellular face of the membrane. Membrane topology prediction programs [41, 42] yielded equivocal results for both proteins. We performed a protease accessibility assay using a *B*. *subtilis* strain engineered to express the sporulation membrane protein SpoIVFA (FA) [43]. FA is an integral membrane protein with a large extracellular domain that is accessible to protease cleavage and served as our positive control. Protoplasts were generated from an exponentially growing culture and then treated with buffer, Proteinase K, or Proteinase K and the detergent N-lauroylsarcosine. As anticipated, FA was accessible to Proteinase K resulting in the loss of its extracellular C-terminal domain, which is recognized by our anti-FA antibody (**Fig 3B**). Consistent with the idea that SweC and SweD are Type II integral membrane proteins with C-terminal intracellular domains, both proteins were inaccessible to protease degradation in protoplasts (**Fig 3B**). The Type II integral membrane protein EzrA and the cytoplasmic protein FtsE served as protease inaccessible controls and were both resistant to Proteinase K in protoplasts. Importantly all four proteins were efficiently degraded by Proteinase K when detergent was included in the reaction. Taken together, these data indicate that the putative coiled-coil and helix-turn-helix domains of SweD and the LysM-like domain on SweC are located in the cytoplasm.

### SweD and SweC require each other for stability

**Fig 4:**
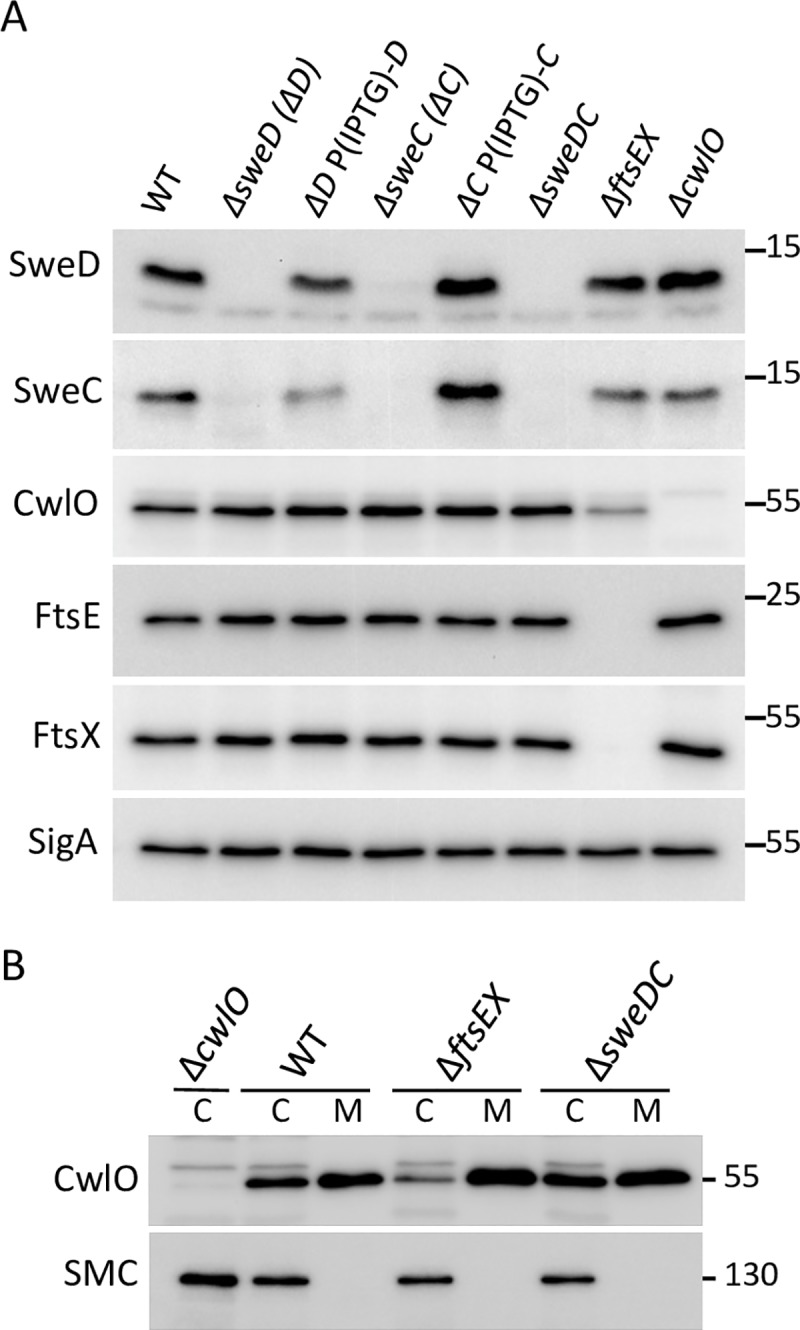
SweD and SweC depend on each other for stability. (**A**) Immunoblot analysis of SweD and SweC in different mutant backgrounds. The indicated strains were grown in LB medium to mid-exponential phase and SweD, SweC, CwlO, FtsE, FtsX and SigA were assessed by immunoblot analysis. SweC levels were almost undetectable in cells lacking SweD (ΔD) but were largely restored when SweD was produced under IPTG control [P(IPTG)-D] from an ectopic locus. Similar results were obtained for SweD in cells lacking SweC (ΔC). By contrast, the levels of FtsE, FtsX, and CwlO were unaffected by the absence of SweD and/or SweC. CwlO levels were partially reduced in the absence of FtsEX. The SigA immunoblot serves to control for loading. The immunoblots shown are from one of three independent experiments. (**B**) Cell-association of CwlO partially depends on FtsEX but not on SweDC. Immunoblot analysis of wild-type (PY79) and cells lacking *cwlO* (BJM54), *ftsEX* (BJM272) and *sweDC* (BYB339). Strains were grown in LB medium to mid-exponential phase and equivalent amounts of whole cell lysate (C) and culture medium (M) concentrated by trichloroacetic acid precipitation were separated by SDS-PAGE. CwlO and the cytoplasmic protein SMC were assessed by immunoblot analysis. The SMC immunoblot serves to control for loading. See Methods for a detailed protocol describing how CwlO was recovered from the medium and the plastic microfuge tube where it bound non-specifically during culture collection. The immunoblots are from one of three independent experiments. Molecular weight markers (in kDa) are indicated.

Proteins that reside in complexes sometimes depend upon each other for stability. Accordingly, to explore whether SweD and SweC interact with each other or with FtsEX, we analyzed whether SweD, SweC, FtsE, FtsX, or CwlO depend on each other for stability. To this end, we compared the levels of all five proteins in wild-type and in strains lacking SweD, SweC, FtsEX, or CwlO. Cells lacking SweD had almost undetectable levels of SweC and reciprocally cells lacking SweC had barely detectable levels of SweD (**Fig 4A**). Expression of either gene in *trans* restored the levels of both proteins, indicating that the effects were not due to polarity or changes in mRNA stability (**Fig 4A**). By contrast, the levels of CwlO and FtsEX were unaffected by the absence of SweD/SweC and SweD/SweC levels were unchanged in the absence of either CwlO or FtsEX. These data are consistent with the idea that SweD and SweC interact with each other and in the absence of either one, the other is susceptible to degradation.

We previously reported that cell association of CwlO does not depend on FtsEX leading us to speculate that an additional factor holds CwlO at the cell surface [25]. This observation, in part, motivated the Tn-Seq screen that identified SweD and SweC. However, we have since discovered that CwlO non-specifically binds to plastic microfuge tubes confounding our original analysis. Using a modified protocol that accounts for this (see Methods) we could detect a decrease in cell-associated (C) CwlO and an increase of the protein in the cultured medium (M) in cells lacking FtsEX compared to wild-type (**Fig 4B**). These data and complementary analysis by Errington and co-workers [22] support the idea that FtsX maintains CwlO at the cell surface as has been observed in other bacteria [26, 27, 29]. Consistent with the topologies of SweD and SweC and the dependencies described above, the amount of surface-associated CwlO was similar in the presence and absence of SweD and SweC (**Fig 4B**).

### The intracellular domains of SweC and SweD are important for function

**Fig 5:**
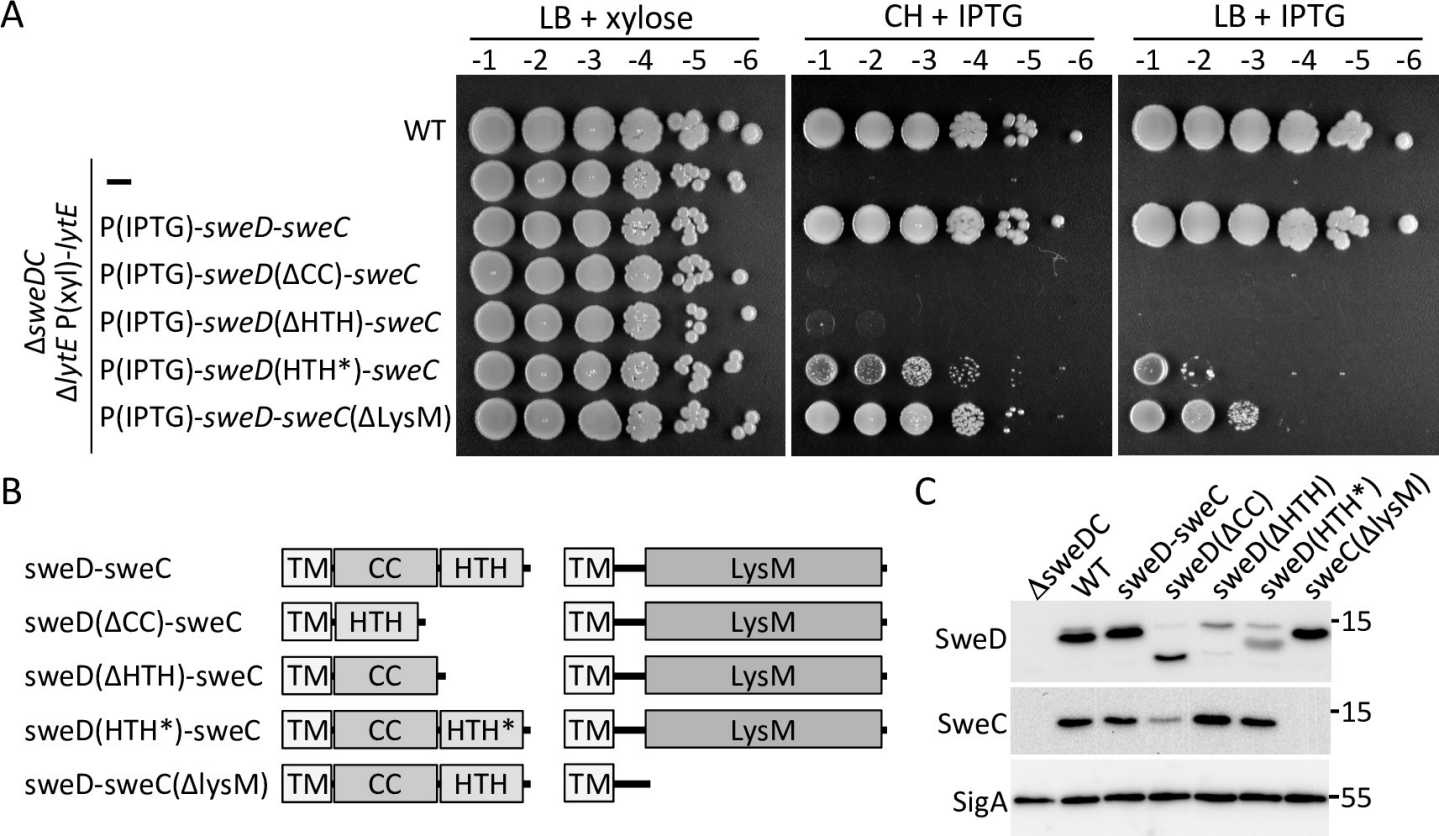
The cytoplasmic domains of SweD and SweC are important for function. (**A**) Spot dilutions of the indicated strains on LB and CH media in the presence of either xylose or IPTG. All strains except wild-type (WT) are Δ*sweDC* Δ*lytE* double mutants and contain a xylose-regulated *lytE* allele. These strains also harbor IPTG-regulated alleles of the *sweDC* operon with the indicated mutations. The strains were grown in LB in the presence of xylose (10 mM) to an optical density of ∼2.0. The cultures were washed twice without inducer, resuspended at an OD_600_ of 1.5, and 10-fold serially diluted. Five microliters of each dilution was spotted onto LB agar plates supplemented with xylose (10 mM) or IPTG (500 μM) and CH agar plates supplemented with IPTG (500 μM). Representative plates from one of three biological replicates are shown. (**B**) Schematic representations of SweD and SweC domain-deletions and point-mutations. HTH* represents amino acid substitutions D104A and V105A in the second helix of the helix-turn-helix motif. (**C**) Immunoblot analysis of the SweDC domain deletion and point mutants. The indicated strains were grown in LB medium to mid-exponential phase. SweD(ΔCC) partially stabilizes SweC. SweD(ΔHTH) is virtually undetectable but fully stabilizes SweC suggesting it is produced but poorly recognized by the anti-SweD antibody. Similarly SweC(ΔLysM) is undetectable but stabilizes SweD. We have not been able to establish the nature of the higher molecular weight band observed in the SweD immunoblot that appears to be an anomalously migrating SweD species but is only present in a subset of lysates and at different levels. The SigA immunoblot serves to control for loading. Molecular weight markers (in kDa) are indicated. Representative immunoblots from one of three independent experiments is shown.

To investigate the contribution of the intracellular domains on SweC and SweD to CwlO activity, a series of domain deletions were generated and tested for their ability to support growth in a LytE depletion strain. Strains lacking either the putative coiled-coil (CC) or HTH domain of SweD largely phenocopied the Δ*sweD* null (**Fig 5A and 5B**) suggesting both domains are critical for function. A strain lacking the LysM homology domain on SweC was impaired for growth upon LytE depletion with a >100-fold plating defect on LB agar (**Fig 5A and 5B**). On defined rich (CH) medium the SweC(ΔLysM) truncation was viable upon LytE depletion albeit with a small colony phenotype. To establish whether these deletion variants were stably produced in vivo, we monitored their levels by immunoblot (**Fig 5C**). Since our antibodies were raised against the soluble intracellular domains of SweD and SweC the immunoblots likely underestimate the levels of these truncations. However, because SweC and SweD require each other for stability we also used the level of the unmodified protein as a proxy for their stability. Based on this analysis, the variants appeared to be produced at levels similar to wild-type with the exception of SweD(ΔCC), which was somewhat reduced (**Fig 5C**). These data indicate that the HTH and CC domains of SweD and the LysM-like domain of SweC are important for function with SweD domains playing more critical roles. Furthermore, these data suggest that if SweD and SweC stabilize each other through interaction then they likely interact via their transmembrane segments.

Finally, we analyzed a SweD variant with mutations in the second helix of the HTH domain that in other HTH DNA binding proteins is involved in interaction with DNA [44]. The mutant was stably produced and maintained SweC levels but was impaired for function (**Fig 5**). ChIP-seq analysis to determine whether SweD binds DNA failed to identify specific binding sites. Specifically, the ratio of sequencing reads between wild-type and *ΔsweDC* mutant samples was ~1 across the entire genome (**S5 Fig**). These data indicate the HTH is important for function but probably not for DNA binding.

### Point mutations in FtsE and FtsX partially bypass the requirement for SweDC

**Fig 6:**
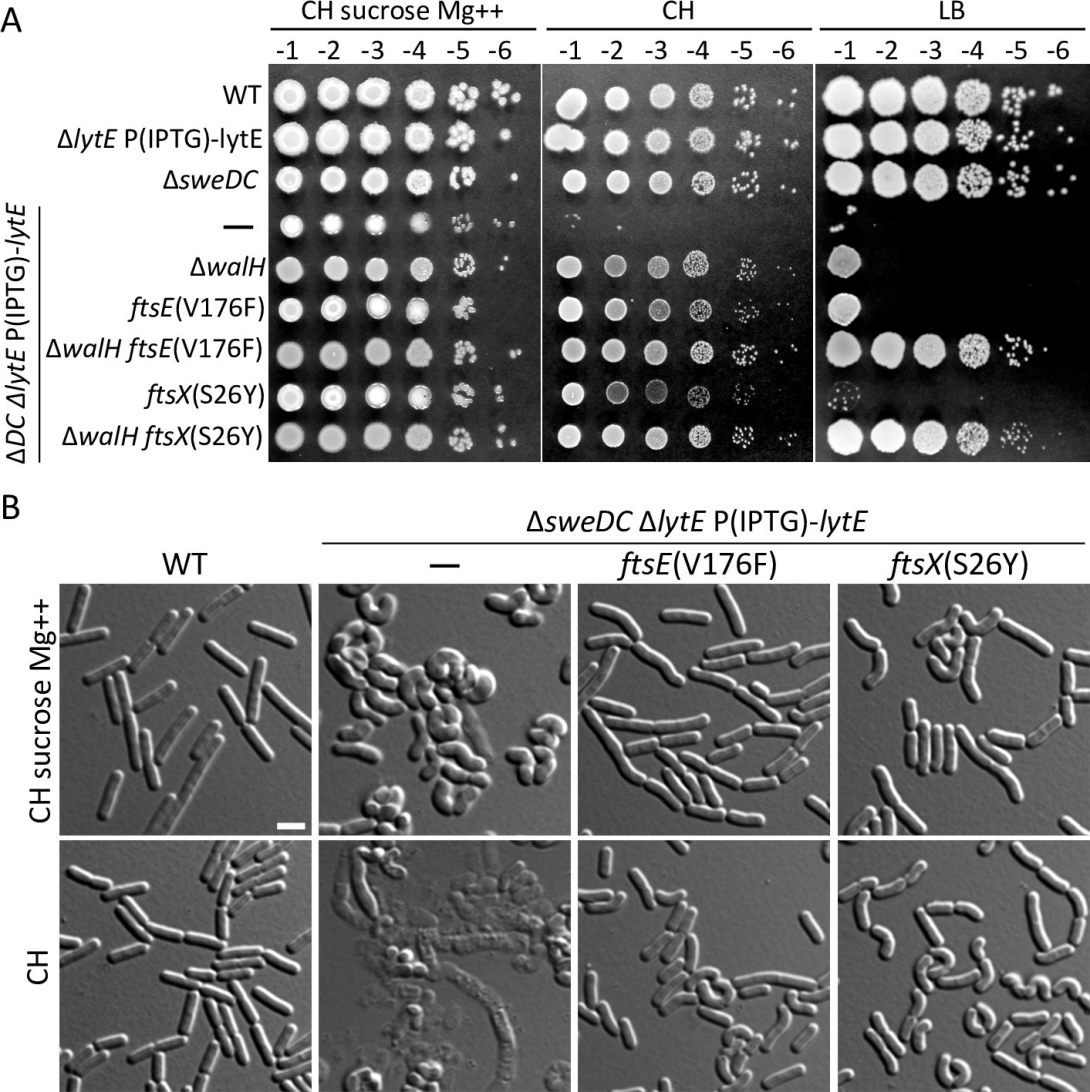
Point mutations in *ftsE* and *ftsX* suppress the Δ*lytE* Δ*sweDC* synthetic lethality. (**A**) Spot dilutions of the indicated strains on permissive and restrictive media. All strains were grown in LB in the presence of IPTG (500 μM) to an optical density of ∼2.0. The cultures were washed twice without inducer, resuspended at an OD_600_ of 1.5, and 10-fold serially diluted. Five microliters of each dilution was spotted onto the indicated agar plates without inducer. The permissive condition contains CH medium supplemented with 0.25 M sucrose and 20 mM MgCl_2_. The Δ*sweDC* (Δ*DC*) Δ*lytE* double mutant, like the parental strain used to select for suppressors, can only grow under permissive conditions. A *walH* deletion or point mutations in *ftsE* or *ftsX* support growth on CH medium lacking IPTG but not LB. Combining Δ*walH* with either point mutation supports growth on LB. Representative plates from one of three biological replicates are shown. (**B**) Cytological analysis of the *ftsEX* suppressors strains under permissive and restrictive conditions. The indicated strains were grown to exponential phase in CH medium in presence of IPTG (500 μM). The cultures were washed twice in CH medium lacking inducer and back-diluted to an OD of 0.01 in permissive and restrictive culture media lacking IPTG. Cells were analyzed using differential interference contrast (DIC) microscopy after 4 generations. Mutations in *ftsE* or *ftsX* restore rod-shape morphology to the Δ*sweDC* Δ*lytE* double mutant when grown under permissive conditions and restored viability and partial rod-shape morphology when grown under restrictive conditions. Scale bar indicates 2 μm. Representative images from one of three independent experiments are shown.

To gain additional insight into the role of SweDC in cell wall elongation, we sought to identify suppressors of the Δ*sweDC* Δ*lytE* double mutant. To this end, we used the LytE depletion strain to screen for conditions in which the double mutant was viable. Mutants with impair cell wall biogenesis are often partially suppressed by growth in hypertonic medium (0.25 M sucrose) supplemented with Mg^2+^ and these conditions were similarly suppressive for cells lacking SweDC and depleted of LytE (**Fig 6A**). The restoration of viability under these conditions is likely due to residual activity of FtsEX-CwlO in the absence of SweDC, because these conditions did not support growth of the Δ*cwlO* Δ*lytE* or Δ*ftsEX* Δ*lytE* double mutants (**S6 Fig**). Next, we generated the Δ*sweDC* Δ*lytE* double mutant in the absence of the IPTG-regulated *lytE* allele using the permissive growth condition. We then grew the cells under permissive conditions and selected for suppressors on LB agar medium. Thirteen independently isolated suppressors were mapped by whole genome re-sequencing (**S7A Fig)**. Eight of the suppressors had loss-of-function mutations in *walH* (*yycH*) encoding a negative regulator of the WalR-WalK two-component signaling pathway [45]. WalR~P is a positive regulator of several PG hydrolases genes including *lytE* and *cwlO* and represses the expression of genes [6, 46, 47] that encode negative regulators of PG hydrolase activity [48, 49]. Among the eight *walH* suppressors, five had second-site missense mutations in either *ftsE* or *ftsX* (**S7A Fig**). The two mutations in *ftsX* mapped to the first (S26Y) and second (S188F) TM segments of the protein (**S7B Fig**). Two mutations in *ftsE* (V176F and T188A) were located in positions predicted to lie adjacent to the nucleotide-binding domain (**S7B Fig**). The third mutation (L90F) was close to the predicted interface between FtsE and FtsX. Three suppressors had missense or small in-frame deletions in *walK* encoding the WalK sensor kinase. One of these mutations has been previously reported to generate a constitutively active allele of an unrelated sensor kinase [50] and we suspect that all three suppressors are hypermorphic alleles. Finally, we identified two independent mutations in *rny* encoding RNase Y, a major endonuclease involved in mRNA decay [51, 52].

The isolations of suppressor mutations in *ftsE* and *ftsX* provided a potential link between SweDC and FtsEX. Accordingly, we focused on these suppressors. To test whether these mutations contributed to the suppression of the Δ*sweDC* Δ*lytE* double mutant, we reconstructed *ftsE*(V176F) and *ftsX*(S26Y) alleles and tested them alone or in combination with a *walH* deletion mutant. As can be seen in Fig 6A, both the *walH* deletion and the *ftsEX* point mutations could suppress the lethality of Δ*sweDC* Δ*lytE* on defined (CH) rich medium but neither were able to support growth on LB. However, combining Δ*walH* with either *ftsEX* allele provided suppression on LB.

Next, we analyzed the morphologies of the suppressors. Depletion of LytE in the absence of SweDC leads to cell lysis in CH medium (**Fig 6B and S8B Fig**). Under permissive growth conditions in the presence of sucrose and Mg^2+^ the cells are fat and curved (**Fig 6B and S8A Fig**). These phenotypes are likely due to residual FtsEX-CwlO activity in the absence of SweDC as we observe similar morphologies in a Δ*lytE* mutant expressing low levels of CwlO when grown under the same permissive condition (**S9 Fig**). We suspect that low-level PG hydrolysis by CwlO results in uneven cell wall elongation leading to the curved cell shape. Importantly, the *ftsE* and the *ftsX* suppressor mutations restored rod-shaped morphologies to the Δ*sweDC* Δ*lytE* double mutant on CH medium supplemented with sucrose and Mg^2+^ and partially suppressed the curved morphologies on CH medium without supplementation (**Fig 6B**). The Δ*walH* mutant also partially restored rod-shape morphology to the Δ*sweDC* Δ*lytE* double mutant on CH medium supplemented with sucrose and Mg^2+^ (**S8B Fig**). Combining the *ftsEX* mutations with Δ*walH* in the Δ*sweDC* Δ*lytE* background further restored wild-type-like morphologies on CH medium with and without supplementation (**S8 Fig**). Collectively, these data support the idea that SweD and SweC function as co-factors of FtsEX in the control of CwlO. The molecular basis for the partial suppression of the Δ*sweDC* Δ*lytE* double mutant by Δ*walH* remains unclear but could be due to the increase in CwlO levels and/or the global change in cell wall hydrolase activity resulting from high WalR~P activity.

### SweD and SweC reside in a complex with FtsX

**Fig 7:**
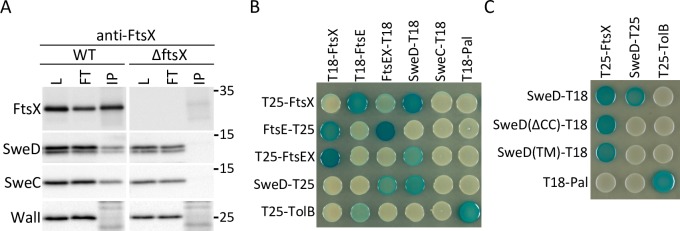
SweD and SweC reside in a complex with FtsX. (**A**) Immunoblot analysis of co-immunoprecipitation assays. Digitonin-solubilized membrane fractions derived from exponentially growing wild-type (WT) (PY79) and Δ*ftsX* (BJM72) mutant cells were incubated with anti-FtsX antisera and precipitated with Protein A Sepharose. The detergent-solubilized fraction prior to immunoprecipitation (load; L), the supernatants after immunoprecipitation (flow through; FT), and the immunoprecipitates (IP) were subjected to immunoblot analysis probing for FtsX, SweD, SweC and a control membrane protein WalI. Molecular weight markers (in kDa) are indicated. Immunoblots are representative of one of two independent experiments. Since the anti-FtsX polyclonal antibodies were not covalently coupled to the Protein A Sepharose the heavy and light chains are present in the IP fractions. The bands and smears detected in the FtsX and WalI immunoblots result from the secondary antibodies recognizing light chains that migrate heterogeneously at ~25 kDa. (**B**) The bacterial adenylate cyclase two-hybrid (BACTH) assay detects an interaction between SweD and FtsX. The BTH101 *E*. *coli* reporter strain containing plasmids with the indicated proteins fused to the T18 or T25 domains of the *Bordetella* adenylate cyclase were spotted on LB(X-gal) indicator plates. Interactions can be detected between FtsE and FtsX, SweD and FtsX, and SweD and itself. T18 and T25 fusions to *E*. *coli* TolB and Pal were used as positive and negative controls. **(C)** Analysis of SweD truncation variants. Interactions can be detected between FtsX and SweD lacking its putative coiled-coil domain (SweDΔCC) and lacking its entire intracellular domain [SweD(TM)]. No interaction was detected between full-length SweD and either of these SweD variants. The BACTH assays were performed in triplicate and photographs of representative agar plates are shown.

The genetic evidence presented thus far place SweD and SweC in the FtsEX-CwlO pathway. To investigate whether SweD or SweC reside in a complex with FtsX, we performed co-immunoprecipitation assays. Crude membrane preparations from wild-type and Δ*ftsX* mutant cells were solubilized with the non-ionic detergent Digitonin. The solubilized membrane proteins were incubated with anti-FtsX polyclonal antiserum and precipitated by Protein A sepharose. The immunoprecipitated material was eluted with SDS sample buffer and analyzed by immunoblot. As can be seen in Fig 7A, the anti-FtsX antibodies efficiently immunoprecipitated FtsX and co-precipitated SweD and SweC but not an unrelated membrane protein WalI (YycI). Importantly, none of these proteins were precipitated from solubilized membrane preparations that lacked FtsX. Thus, these data indicate that SweD and SweC reside in a membrane complex with FtsX.

To investigate whether FtsE or FtsX interact directly with either of these factors we used the Bacterial Adenylate Cyclase Two Hybrid (BACTH) system. We generated fusions with complementary fragments (T18 and T25) of *Bordetella pertussis* adenylate cyclase to FtsE, FtsX, SweD and SweC. As anticipated, we detected positive interactions between FtsE and FtsX with several but not all fusions and with the positive control TolB and Pal (**Fig 7B**). A weak interaction was also observed between TolB and FtsE. This false positive highlights the limitations of the two-hybrid assay and the importance of interpreting positive interactions cautiously. The assay also revealed positive interactions between FtsX and SweD with two distinct fusion pairs and an interaction between SweD and itself. Furthermore, a SweD variant lacking its coiled-coil (CC) domain retained the ability to interact with FtsX but not with full-length SweD (**Fig 7C**), suggesting that the CC domain functions in SweD dimerization. Finally, a SweD variant lacking its entire intracellular domain [SweD(TM)] was unaffected in its ability to interact with FtsX (**Fig 7C**), suggesting that SweD interacts with FtsX via its TM segment. Despite our in vivo data that SweC and SweD depend on each other for stability and our co-immunoprecipitation assay that identified SweC in a complex with FtsX, we were unable to detect an interaction between SweC and either of these two proteins in the two-hybrid assay.

## Discussion

**Fig 8:**
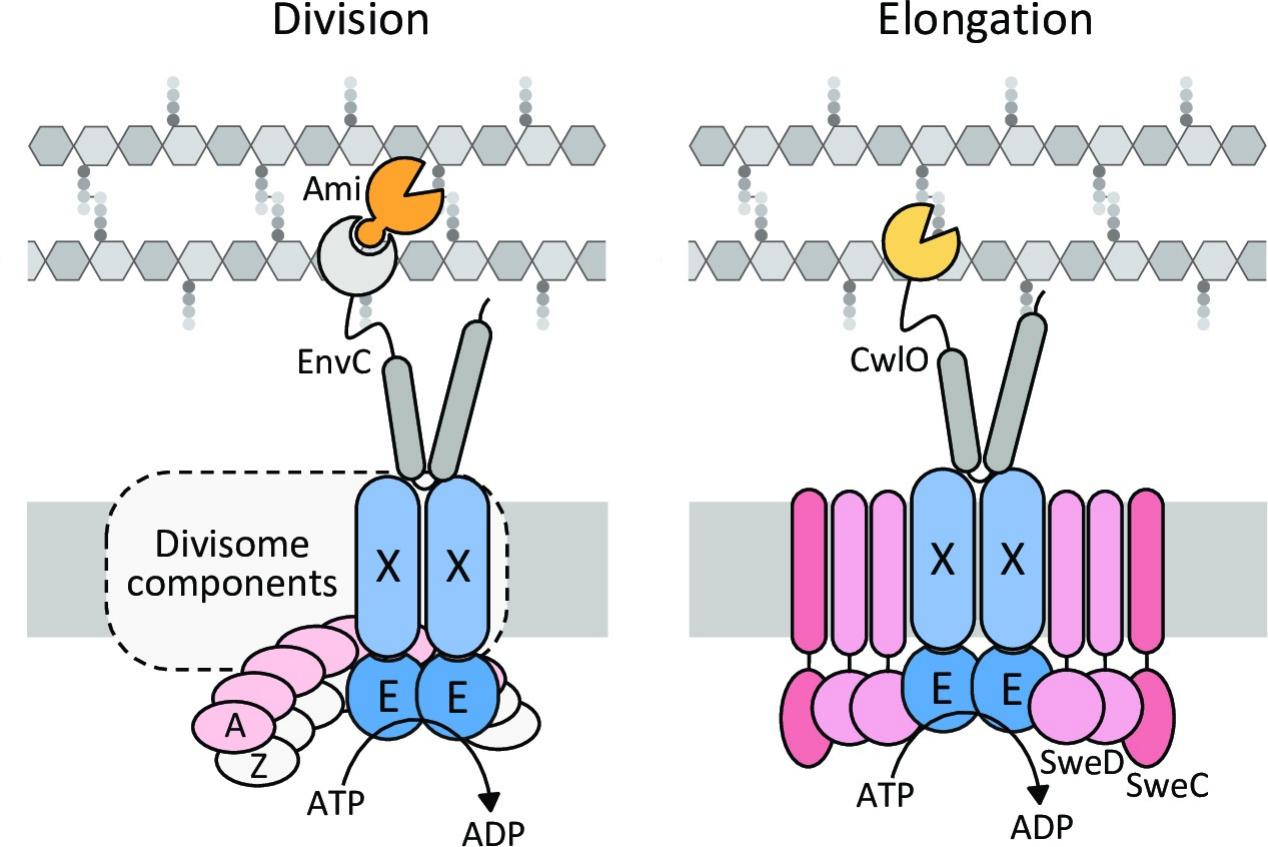
FtsEX complexes employ distinct co-factors to regulate PG hydrolysis during cell division and cell wall elongation. Schematic models comparing the *E*. *coli* FtsEX-EnvC-Amidase complex that functions during division (left) with the *B*. *subtilis* SweDC-FtsEX-CwlO elongation hydrolase complex (right). In *E*. *coli*, FtsE (E)—FtsX (X) complex promotes divisome assembly [32, 33] and regulates septal cell wall cleavage by the amidases AmiA and AmiB (Ami) via the coiled-coil domain-containing regulator EnvC [27, 31]. FtsEX has been found to interact with FtsZ (Z) [81] and FtsA (A) [32] and FtsEX ATPase activity is required for both PG synthesis [32, 34] and amidase activity [27]. SweD and SweC reside in a membrane complex with FtsEX and function as co-factors in regulating the D,L-endopeptidase activity of CwlO. Based on ribosome profiling and our two-hybrid analysis, the stoichiometry of the SweD-SweC-FtsE-FtsX complex is proposed to be 4:2:2:2. Our analysis suggests that SweDC regulate the FtsEX ATPase activity. Genetic evidence suggests that the hydrolase complex functions in the same pathway as the peptidoglycan elongation machinery (not depicted) [22]. Whether this is direct or indirect is currently unknown.

Altogether our data indicate that SweD and SweC reside in a multimeric membrane complex with FtsX and function as essential co-factors of FtsEX in its control of CwlO activity during cell wall elongation (**Fig 8**). Ribosome profiling from exponentially growing *B*. *subtilis* cells suggests that the levels of SweC are similar to FtsE and FtsX while SweD levels are ~2-fold higher [53]. Based on these data and our two-hybrid analysis, we hypothesize that the SweD-SweC-FtsE-FtsX complex has a stoichiometry of 4-2-2-2. Finally, our data indicate that both SweD and SweC have conserved intracellular domains that are important for function and could therefore play roles in maintaining or regulating the ATPase activity of FtsE; directly sensing a signal to modulate cleavage activity; and/or linking the PG hydrolase complex to PG synthesis machinery. The identification of suppressor mutations adjacent to the ATPase domain of FtsE that partially bypass the requirement for SweDC suggests that these co-factors could indeed help maintain and/or control cycles of ATP hydrolysis. However, attempts to reconstitute ATPase activity of FtsE in vitro have thus far been unsuccessful making it difficult to directly test this model. It is also noteworthy that our data indicate that the LysM-like domain on SweC resides in the cytosol rather than facing the cell wall. Since these domains bind polysaccharides, an attractive model is that this domain functions to coordinate PG hydrolase activity with cell wall synthetic capacity by monitoring cytosolic PG precursors. In vitro reconstitution of the complex in the future will allow us to explore this and related models for SweDC function.

A connection between the FtsEX-CwlO PG hydrolase complex and the cell wall elongation machinery (called the Rod complex) has been proposed previously [22] but the evidence remains incomplete. Cells lacking LytE but not FtsEX or CwlO were found to have a synthetic growth defect when the actin-like protein Mbl was depleted. Since Mbl functions as a scaffold for the Rod complex, these data suggest that CwlO, FtsEX, Mbl and by extension the rest of the cell wall elongation machinery are in the same genetic pathway [22]. We detect similar growth defects when Mbl is depleted in a Δ*lytE* mutant and the absence of significant growth defects when Mbl is depleted in strains lacking FtsEX, CwlO, or SweDC (**S10 Fig**). However, we found that a functional SweD-GFP fusion (**S11 Fig**) lacked the dynamic behavior of the Rod complex, which was shown to move in a directed and circumferential manner around the long axis of cell [54–56]. These data suggest that the SweDC-FtsEX-CwlO complex is unlikely to be a core component of the cell wall synthetic machinery but does not exclude the possibility that the two complexes transiently interact and influence each other's activity. Consistent with this idea, formaldehyde crosslinking of *B*. *subtilis* cells followed by affinity purification of Mbl and Mass Spectrometry identified FtsX and SweC in crosslinked complexes with Mbl and separately FtsE and FtsX in crosslinked complexes with MreB [57]. However, >65 proteins were identified in each of the crosslinked complexes making it difficult to draw strong conclusions from these findings. Finally, we note that the integral membrane protein RodZ, a core member of the Rod complex, has an intracellular HTH motif like SweD [58–60]. In the case of RodZ, this motif functions to position an adjacent helix such that it can interact with MreB [61]. Although we have shown that the HTH motif on SweD is important for function this domain lacks the analogous interaction helix found in RodZ. Furthermore, we did not observe an interaction between Mbl and SweD in the bacterial two-hybrid assay. Thus, it remains an open question whether the PG hydrolase activity of the SweDC-FtsEX-CwlO complex is coordinated with cell wall synthesis mediated by the Rod complex.

We note that gram-positive bacteria like *Bacillus subtilis* are thought to synthesize their cell wall via an inside to outside mechanism in which newly synthesized layers adjacent to the cell membrane are not initially load bearing [3]. Only as these layers migrate outward during cell growth do they experience stress. It is these more distal load-bearing layers that are likely to be the target of CwlO and LytE. Thus, the activity of these D,L-endopeptidases would not necessarily need to be directly coordinated with PG synthesis as has been proposed for gram-negative bacteria [62].

Finally, we return to FtsEX. This noncanonical ABC transporter is the most broadly conserved regulator of PG hydrolases. In many cases this complex is intimately associated with the divisome where it controls cell wall cleavage during cytokinesis and potentially coordinates PG hydrolysis with septal PG synthesis (**Fig 8**). Here, we establish that two co-factors SweD and SweC are critical for FtsEX function outside the divisome during cell wall elongation. Homologs of SweD and SweC are present in Bacilliaceae, Lactobacillales, and Listeriaceae species, where we propose that, like the *B*. *subtilis* co-factors, they enable FtsEX to control elongation PG hydrolases. Interestingly, work from the Bernhardt lab has uncovered a distinct set of factors that function with FtsEX in *Corynebacterium glutamicum*, an actinobacterium that undergoes cell separation through a mechanically driven process called V snapping [88]. These two membrane proteins (SteA and SteB) are required for the FtsEX-RipC PG hydrolase complex to promote for V snapping. Thus, the identification of SweDC and SteAB reveal that the broadly conserved FtsEX regulatory module works with distinct co-factors to control PG hydrolases required for cell division, cell separation, and cell wall elongation.

## Materials and methods

### General methods

All *B*. *subtilis* strains were derived from the prototrophic strain PY79 [63]. Unless otherwise indicated, cells were grown in LB or defined rich (casein hydrolysate, CH) medium at 37°C. Insertion-deletion mutations were generated by isothermal assembly [64] of PCR products followed by direct transformation into *B*. *subtilis*. Tables of strains, plasmids and oligonucleotide primers and a description of strain and plasmid construction can be found online as supplementary data (**S1, S2 and S3 Tables, and S1 Text**).

### Transposon insertion sequencing

Transposon insertion sequencing (Tn-seq) was performed as described previously [65–67]. Libraries of >100,000 independent transposants were separately generated in wild-type and a Δ*lytE* mutant. Genomic DNA was extracted from each and digested with MmeI, followed by adapter ligation. Transposon-chromosome junctions were amplified by PCR (17 amplification cycles). PCR products were pooled, gel-purified, and sequenced on the Illumina HiSeq platform using TruSeq reagents (Tufts University TUCF Genomics facility). Reads were mapped to the *B*. *subtilis* 168 genome (NCBI NC_000964.3), tallied at each TA site, and genes in which reads were statistically underrepresented were identified using the Mann Whitney *U* test and by visual inspection using Sanger Artemis Genome Browser and Annotation tool [68].

### Immunoblot analysis

Immunoblot analysis was performed as described previously [69]. Briefly, 1ml of culture was collected and resuspended in lysis buffer [20 mM Tris pH 7.0, 10mM MgCl_2_ and 1mM EDTA, 1 mg/ml lysozyme, 10 μg/ml DNase I, 100 μg/ml RNase A, 1 mM PMSF, 1 μg/ml leupeptin, 1 μg/ml pepstatin] to a final OD_600_ of 10 for equivalent loading. The cells were incubated at 37°C for 10 min followed by addition of an equal volume of sodium dodecyl sulfate (SDS) sample buffer [0.25 M Tris pH 6.8, 4% SDS, 20% glycerol, 10 mM EDTA] containing 10% 2-Mercaptoethanol. Samples were heated for 15 min at 65°C prior to loading. Proteins were separated by SDS-PAGE on 10% (for SMC), 12.5% (for FtsE, FtsX, CwlO, SigA, EzrA, ScpB, SpoIVFA, and WalI) or 20% (for SweD and SweC) polyacrylamide gels, electroblotted onto Immobilon-P membranes (Millipore) and blocked in 5% nonfat milk in phosphate-buffered saline (PBS) with 0.5% Tween-20. The blocked membranes were probed with anti-SweD (1:10,000), anti-SweC (1:10,000), anti-EzrA (1:10,000) [39], anti-SpoIVFA (1:10,000) [70], anti-SMC (1:10,000) [71], anti-SigA (1:10,000) [72], anti-ScpB (1:10,000) [73], anti-FtsE (1:20,000) [25], anti-FtsX (1:10,000) [25], anti-CwlO (1:10,000) [25], anti-WalI (YycI) [74] diluted into 3% BSA in 1x PBS with 0.05% Tween-20. Primary antibodies were detected using horseradish peroxidase-conjugated goat anti-rabbit IgG (BioRad) and the Super Signal chemiluminescence reagent as described by the manufacturer (Pierce). Signal was detected using a Bio-Techne FluorChem R System.

### Fluorescence microscopy

Fluorescence microscopy was performed on a Nikon Ti microscope equipped with Plan Apo 100x/1.4NA phase contrast oil objective and a CoolSnapHQ^2^ camera. Cells were immobilized using 1.5% agarose pads containing CH medium. Membranes were stained with TMA-DPH (50μM) (Molecular Probes). Exposure times were 400 ms and 800 ms for TMA-DPH and mCherry, respectively. Quantitative image analysis was performed using Oufti [75]. Meshes were created using the cytoplasmic mCherry images. The length of the long axis of >350 cells was determined and the mean cell length was calculated. Images were cropped and adjusted using MetaMorph software (Molecular Devices). Final figures were prepared in Microsoft PowerPoint.

### Protein purification and antibody production

Recombinant proteins were expressed in *E*. *coli* strain BL21 (DE3). Strains were grown in 500 mL of auto-induction Overnight Express Instant TB Medium (Novagen) supplemented with 100 μg/ml ampicillin at 22°C. After 16 h, cultures were subjected to centrifugation at 10,000 × *g* for 10 min. Cell pellets were resuspended in 15mL Lysis Buffer (20 mM Tris pH 7.5, 300 mM NaCl, 5 mM imidazole, 10% glycerol, 0.1 μM Dithiothreitol) and Complete EDTA-free protease inhibitors (Roche) and lysed via passage through a French press. Cell lysates were clarified by centrifugation at 10,000 X *g* for 10 minutes at 4°C. Clarified lysates were mixed with 0.5 mL of Ni-NTA agarose resin (Qiagen) and incubated for 2 hours at 4°C. The mixture was loaded onto a BioRad column, washed with 10 mL Buffer A (20 mM Tris pH 7.5, 300 mM NaCl, 5 mM imidazole, 10% glycerol, 0.1 μM Dithiothreitol). His_6_-SUMO fusion proteins were eluted with Buffer B (20 mM Tris pH 7.5, 300 mM NaCl, 200 mM imidazole, 0.1 μM Dithiothreitol). Eluates were pooled and dialyzed into 20 mM Tris pH 7.5, 300 mM NaCl, 10% glycerol, 0.1 μM Dithiothreitol at 4°C. The dialysates were incubated with His_6_-Ulp1 protease overnight on ice. Reactions were mixed with 0.5 mL Ni-NTA agarose and loaded onto BioRad columns. Flow through fractions containing cleaved (untagged) proteins were collected and used to generate rabbit polyclonal antibodies (Covance).

### Protease accessibility assay

25 mL of exponentially growing cells were collected, washed, and resuspended in 5mL 1X SMM buffer (0.5 M sucrose, 20 mM MgCl_2_, 20 mM maleic acid pH 6.5) [76] supplemented with Lysozyme (4 mg/ml). Cells were incubated for 30 minutes at RT with gentle agitation. Protoplast formation was monitored by microscopic observation on 2% 1X SMM-agarose pads. When >95% of the cells were protoplasted they were collected by centrifugation (5 Krpm) and resuspended in 1 mL of 1X SMM buffer and distributed into three microfuge tubes. Protoplasts were incubated with: 1X SMM buffer alone or with Proteinase K (NEB, 50 ug/ml final), or Proteinase K and Sodium-lauroyl-sarcosinate (1%) for 15 min at room temperature. Proteinase K was inactivated by the addition of 2X sample buffer [0.25 M Tris pH 6.8, 4% SDS, 20% glycerol, 10 mM EDTA 10% 2-Mercaptoethanol] supplemented with 2 mM PMSF and immediately incubated at 100°C for 10 minutes. Reactions were analyzed by immunoblot.

### CwlO fractionation

CwlO binds non-specifically to plastic microfuge tubes but can be recovered from them with SDS sample buffer. To monitor the amount of cell-associated and released CwlO, 1.5 mL of a mid-exponential phase culture (grown in LB) was collected in a plastic microfuge tube and centrifuged for 10 min at 3,000 × *g*. The culture supernatant was transferred to a fresh microfuge tube and the secreted proteins were precipitated by the addition of 15% trichloroacetic acid. The cell pellet was gently resuspended in 50 μL lysis buffer [20 mM Tris pH 7.0, 10mM MgCl_2_ and 1mM EDTA, 10 μg/ml DNase I, 100 μg/ml RNase A, 1 mM PMSF, 1 μg/ml leupeptin, 1 μg/ml pepstatin] and transferred to a fresh microfuge tube followed by the addition of lysozyme (1 mg/ml). A whole cell lysate was then prepared as described in the immunoblot protocol above. The microfuge tube used to collect cells and culture medium was washed twice with 1 mL LB to remove any remaining cells. The proteins bound non-specifically to the tube were then released with SDS sample buffer. The sample buffer with released proteins was then used to resuspend the TCA-precipitated proteins from the culture supernatant. Equivalent amounts of cell lysate (C) and culture medium (M) were resolved by SDS-PAGE and CwlO and SMC were analyzed by immunoblot.

### Suppressor selection

The Δ*sweDC*::*kan ΔlytE*::*cat* double mutant was constructed under permissive growth conditions (CH agar supplemented with 20 mM MgCl_2_ and 0.25 M sucrose). Three independent clones (BYB366, BYB367, BYB368) were grown to early stationary phase under permissive conditions at 37°C. Cells were washed twice in fresh LB and plated under permissive and restrictive conditions (LB agar) at 37°C. The suppressor frequency was ~10^−7^. Genomic DNA from 13 suppressors was prepared for whole genome sequencing using a modified Nextera library preparation protocol [77]. DNA concentrations were determined using the Qubit dsDNA HS Assay Kit and fragment sizes were determined using a High Sensitivity D1000 screen tape run on an Agilent 4200 TapeStation system. Sequencing was performed using a MiSeq Kit v6, with the Miseq System (Illumina). Reads were mapped using CLC Genomics Workbench software (Qiagen).

### Co-immunoprecipitation from detergent-solubilized membrane fractions

Co-IPs were performed as described previously [78]. Briefly, 150 mL cultures of wild-type and the Δ*ftsX* mutant were harvested at an OD_600_ of 0.5 washed twice with 1X SMM (0.5 M sucrose, 20 mM MgCl_2_, 20 mM maleic acid pH 6.5) at room temperature. Cells were resuspended in 1:10 volume 1XSMM and protoplasted with lysozyme (0.5 mg/mL final) for 30 minutes. Protoplasts were collected by centrifugation and disrupted by osmotic lysis with 3 ml hypotonic buffer (Buffer H) (20 mM Hepes pH 8, 200 mM NaCl, 1 mM Dithiothreitol) with protease inhibitors: 1 mM phenylmethylsulfonyl fluoride and EDTA-free protease inhibitor cocktail complete (Roche). MgCl_2_ and CaCl_2_ were added to 1 mM and lysates were treated with DNAse I (10 μg/ml) (Sigma-Aldrich) and RNAse A (20 μg/ml) (USB) for 1 h on ice. The membrane fraction was separated by ultracentrifugation at 100,000 × *g* for 1 h at 4°C. The supernatant was carefully removed, and the membrane pellet was dispersed in 400 μL of Buffer G (Buffer H with 10% glycerol). Crude membranes were aliquoted and flash-frozen in N_2_(l). 200 μL crude membranes were diluted 5-fold with Buffer S (Buffer H with 20% glycerol and 100 μg/ml lysozyme), and membrane proteins were solubilized by the addition of the nonionic detergent digitonin (Sigma) to a final concentration of 0.5%. The mixture was rotated at 4°C for 1 h. Soluble and insoluble fractions were separated by ultracentrifugation at 100,000 × *g* for 1 h at 4°C. The soluble fraction from the digitonin-treated membrane preparation (the load) was mixed with 4 μl of crude anti-FtsX antiserum [25] and rotated for 3 h at 4°C. The mixture was added to 25 μL of Protein A Sepharose resin (GE Healthcare) and rotated for 1 h at 4°C. The resin was pelleted at 5 Krpm, and the supernatant (the flow-through) was collected. The resin was washed four times with 0.4 mL of Buffer S + 0.5% digitonin. Immunoprecipitated proteins were eluted by the addition of 50 μl of sodium dodecyl sulfate (SDS) sample buffer (0.25 M Tris, pH 6.8, 6% SDS, 10 mM EDTA, 20% glycerol) and heated for 15 min at 50°C. The resin was pelleted, and the supernatant (the IP) was transferred to a fresh tube and 2-mercaptoethanol was added to a final concentration of 10%. The load, flow-through, and immunoprecipitate were analyzed by immunoblot.

### Bacterial two-hybrid assay

The Bacterial Adenylate Cyclase-based Two Hybrid (BACTH) system was used as previously described ([79, 80]. Briefly, pairs of proteins were fused to the complementary fragments (T18 and T25) of the *Bordetella pertusis* adenylate cyclase. After co-transformation into BTH101, independent transformants were inoculated in LB medium supplemented with ampicillin (100 μg/mL), kanamycin (30 μg/mL) and 0.5 mM isopropyl-β-thio-galactoside (IPTG). Cells were grown at 30°C overnight and spotted on LB agar plates supplemented with ampicillin (100 μg/mL), kanamycin (30 μg/mL), IPTG (0.25 mM) and 5-bromo-4-chloro-3-indolyl-β-D-galactopyrannoside (X-Gal) (20 mg/mL). Plates were photographed after incubation at 30°C for 16 hours.

### Chromatin immunoprecipitation

Chromatin immunoprecipitation (ChIP) was performed as described previously [69]. Briefly, cells were crosslinked using 3% formaldehyde for 30 min at room temperature and then quenched, washed, and lysed. Chromosomal DNA was sheared to an average size of 250 bp by sonication using a Qsonica Q800 water bath sonicator. The lysate was then incubated overnight at 4°C with anti-SweD antisera, and was subsequently incubated with Protein A-Sepharose resin (GE HealthCare) for 1 hr at 4°C. After washes and elution, the immunoprecipitate was incubated at 65°C overnight to reverse the crosslinks. The DNA was further treated with RNase A, Proteinase K, extracted using Phenol-Chloroform, resuspended in 50 μl EB and used for library preparation with the NEBNext Ultra kit (E7370S) and sequenced using the Illumina MiSeq platform.

The sequencing reads from wild-type and *ΔsweDC* ChIP samples were mapped to the *B*. *subtilis* PY79 genome (NCBI Reference Sequence: NC_022898.1) using CLC Genomics Workbench (CLC bio, QIAGEN). Subsequent normalization, plotting, and analyses were done using R plots as follows. Samples were first normalized to the total number of reads. Then the ratio of ChIP signal in wild-type relative to *ΔsweDC* was calculated and plotted in S4A Fig. The data were plotted in 1 kb windows. For S4B Fig, ChIP signals of wild-type and *ΔsweDC* between 2390kb and 2400kb were plotted.

## Supporting information

S1 Fig Characterization of the Δ*sweD* and Δ*sweC* single mutants. (**A**) *sweD* and *sweC* are synthetically lethal with *lytE* but not *cwlO* or *ftsEX*. Spot dilutions of the indicated strains in the presence and absence of inducer. All strains were grown in the presence of IPTG (500 μM) to an optical density of ∼2.0. The cultures were washed twice without inducer, resuspended at an OD_600_ of 1.5, and 10-fold serially diluted. Five microliters of each dilution were spotted onto LB agar plates with and without IPTG (500 μM). Representative plates from one of three biological replicates are shown. (**B**) Cells lacking SweD or SweC have similar morphological defects. Exponentially growing cells were stained with the lipophilic dye TMA-DPH and examined by fluorescence and phase-contrast microscopy. The representative images shown are from one of three independent experiments. Scale bar indicates 2 μm.
(TIF)

S2 Fig SweD and SweC are conserved in a subset of firmicutes. (**A**-**B**) Amino acid alignments of a subset of SweD and SweC orthologs from Bacilliaceae (Bac), Lactobacillales (Lacto), and Listeriaceae (Lister) family members. SweC orthologs were identified by PSI-BLAST and were analyzed manually for the presence of a putative N-terminal TM segment using TMHMM [42] and a C-terminal LysM-like domain using HHPred [82]. SweD orthologs were identified by analyzing the gene upstream of *sweC* for the presence of an N-terminal TM segment and a coiled-coil domain using COILS [83]. Alignments were generated using the Clustal Omega server [84] and shaded using BOXSHADE (https://embnet.vital-it.ch/software/BOX_form.html). Identical (black) and conserved (grey) residues are highlighted. Entrez database gene names are: *yqzD* (BSU24930), N288_18180, GTNG_2391, WP_010173423, Aflv_0885, CUB26036, HMPREF9257_0979, YqzD, SAMN04488506_1998, SAMN04488559_10551, SAMN04489868_11726, HMPREF0446_00721, LMO1334, LIN1371, LWE1349, HMPREF0556_12475, LIV_1285, *yqzC* (BSU24940), N288_18185, GTNG_2392, WP_010173421, Aflv_0884, CUB26039, HMPREF9257_0980, YqzC, SAMN04488506_1999, SAMN04488559_10550, SAMN04489868_11725, HMPREF0446_00720, LMO1333, LIN1370, LWE1348, HMPREF0556_12476, LIV_1284.
(TIF)

S3 Fig Quantitative analysis of cell length during depletion of SweDC or CwlO in the presence and absence of LytE. (**A**) mCherry images from the time-course experiment presented in Fig 2B were analyzed using Oufti [75] to assess cell length at the indicated time points after removal of IPTG. Strains were wild-type (WT) (BDR2649), Δ*lytE* (BYB373), Δ*sweDC*, P(IPTG)-*sweDC* (BYB360) and Δ*lytE*, Δ*sweDC*, P(IPTG)-*sweDC* (BYB362). >350 cells were analyzed at each time point. (**B**) mCherry images from the time-course experiment presented in Fig 2C were analyzed using Oufti to assess cell length at the indicated time points. Strains analyzed were wild-type (WT) (BDR2649), Δ*cwlO*, P(IPTG)-*cwlO* (BYB265) and Δ*lytE*, Δ*cwlO*, P(IPTG)-*cwlO* (BYB279). >350 cells were analyzed at each time point.
(TIF)

S4 Fig The curved morphologies of the Δ*sweDC* mutant depend on CwlO. Cytological analysis comparing the terminal phenotypes of strains depleted of LytE in the absence of *sweDC*, *cwlO*, or both. The indicated strains (BYB1181, BYB1185, BYB1447, BYB1446) were grown to exponential phase in CH medium supplemented with IPTG (500 μM), washed twice with medium lacking inducer, back-diluted to an OD_600_ of 0.05 (BYB1181, BYB1446) or 0.1 (BYB1185, BYB1447) and grown to mid-exponential phase in the absence of inducer. Cells were examined by fluorescence microscopy every 30 min. Representative images 90 min after the removal of IPTG are shown. The Δ*sweDC* mutant was visualized 180 minutes after LytE shut-off because more time was required to reach the terminal phenotype. Phase contrast (phase), cytoplasmic mCherry fluorescence, and an overlay (merge) are shown. The representative images are from one of three independent experiments. Scale bar indicates 2 μm. The apparent differences in chaining of the various mutants in the images presented do not reflect cell separation defects. In larger fields of cells there was no discernable difference in cell separation among the strains presented.
(TIF)

S5 Fig SweD is not enriched at specific genomic loci. **(A)** The ratio of normalized ChIP-seq reads obtained in wild-type and the *ΔsweDC* mutant plotted across the genome. ChIP-seq using anti-SweD antibodies was performed on wild-type and *ΔsweDC* cells. Sequencing reads from both samples were normalized to the total number of reads for each sample. The ratio of normalized WT to *ΔsweDC* reads was plotted in 1 kb bins. The peak at 2394 kb overlaps the *sweDC* locus. **(B)** Zoom-in to the *sweDC* locus. ChIP-seq reads of wild-type (black line) and *ΔsweDC* mutant (blue line) were plotted at genome location 2390–2400 kb. The ChIP-seq plots of both sample show similar profiles, except at the *sweDC* locus explaining the high ChIP-seq ratio at this position in (A).
(TIF)

S6 Fig Hypertonic medium supplemented with Mg^2+^ does not support growth of Δ*cwlO* or Δ*ftsEX* upon depletion of LytE. Spot dilutions of the indicated strains (PY79, BYB32, BYB35, BYB36, BYB515) in the presence and absence of inducer. All strains were grown in the presence of IPTG (500 μM) to an optical density of ∼2.0. The cultures were washed twice without inducer, resuspended at an OD_600_ of 1.5, and 10-fold serially diluted. Five microliters of each dilution was spotted onto CH plates supplemented with 20 mM MgCl_2_ and 0.25 M sucrose with and without inducer. Representative plates from one of three biological replicates are shown.
(TIF)

S7 Fig Suppressors of the Δ*sweDC* Δ*lytE* double mutant. (**A**) Mutations in the Δ*sweDC* Δ*lytE* suppressor strains identified by whole genome re-sequencing. Most suppressors had loss-of-function mutations in *walH* encoding a negative regulator of the WalK sensor kinase [45]. Five of these had second-site mutations in *ftsE* or *ftsX*. All five mutations in *ftsE* or *ftsX* were separately reconstructed and found to suppress the lethality of the Δ*sweDC* Δ*lytE* double mutant on defined (CH) rich medium in the presence of wild-type *walH*. Three suppressors had missense mutations in *walK* that are predicted to cause constitutive signaling [50, 85]. Two suppressors had mutations in *rny* encoding Rnase Y. The *cwlO* mRNA was shown to be stabilized in the absence of RNAse Y [86]. However, ~2-fold over-expression of CwlO was not sufficient to suppress the Δ*sweDC* Δ*lytE* mutant. (**B**) Homology model of *B*. *subtilis* FtsEX generated using the SWISS-MODEL server [87]. The structure of the *Aggregatibacter actinomycetemcomitans* ABC transporter MacB (PDB: 5LIL) [28] and that of the large extracellular loop of *Mycobacteria tuberculosis* FtsX (PDB: 4N8O) [23] were used as templates. The residues in FtsX (S26 and S188) and in FtsE (L90, V176, and T188) that were identified in the suppressor selection are highlighted in pink and red, respectively. Boxed regions highlight the TM segments in FtsX that contain the two serine residues (pink) that suppressed when substituted (top) and an FtsE monomer with ATP modeled into the nucleotide binding pocket (bottom). Walker A and B motifs are shown in light and dark blue. The three residues in FtsE (red) that suppressed when substituted are indicated.
(TIF)

S8 Fig Suppression of Δ*sweDC* Δ*lytE* by Δ*walH*, point mutations in *ftsE* or *ftsX*, or the combination. Cytological analysis of the suppressors strains under permissive (**A**) and restrictive conditions (**B**). The indicated strains were grown to exponential phase in CH medium in the presence of IPTG (500 μM). The cultures were washed twice with medium lacking inducer, back-diluted to an OD_600_ of 0.05 in permissive or restrictive culture medium (CH medium supplemented with 20 mM MgCl_2_ and 0.25 M sucrose and CH medium alone, respectively). Cells were immobilized on 2% agarose CH (20 mM MgCl_2_ and 0.25 M Sucrose) or agarose CH pads and observed using differential interference contrast (DIC) microscopy. Representative images from one of three biological replicates are shown. Scale bar indicates 2 μm.
(TIF)

S9 Fig Low level expression of CwlO in the absence of LytE phenocopies depletion of LytE in the absence of SweDC. Comparison of cell morphologies in strains lacking SweDC and depleted of LytE (BYB515) or expressing low levels of CwlO in the absence of LytE (BYB287). Both strains were grown under permissive conditions (CH medium supplemented with 0.25 M sucrose and 20 mM MgCl_2_). Representative DIC images are shown. Cells were grown to exponential phase in CH medium supplemented with IPTG (500 μM), washed twice with medium lacking inducer, back-diluted to an OD_600_ of 0.02 under permissive growth conditions in the presence (BYB287) or absence (BYB515) of 15 μM IPTG. After five generations, cells were immobilized on 2% agarose, 20 mM MgCl_2_ and 0.25 M sucrose CH pads and observed using differential interference contrast (DIC) microscopy. The representative images shown are from one of three biological replicates. Scale bar indicates 2 μm.
(TIF)

S10 Fig *lytE* and *mbl* are a synthetically lethal pair. Spot dilutions of the indicated strains in the presence and absence of inducer. All strains were grown in the presence of IPTG (500 μM) to an optical density of ∼2.0. The cultures were washed twice without inducer, resuspended at an OD_600_ of 1.5, and 10-fold serially diluted. Five microliters of each dilution were spotted onto LB agar plates with and without IPTG (500 μM) and LB agar plates supplemented with 5 mM MgCl_2_, a semi-permissive condition for cells lacking Mbl. Cells depleted of Mbl on LB agar in the presence or absence of 5 mM MgCl_2_ have a slow-growth phenotype. The growth defect is modestly enhanced to a similar degree in the absence of CwlO, FtsEX, or SweDC. However, in the absence of LytE, depletion of Mbl results in 4-log plating defect as reported previously [22]. These data provide evidence that Mbl is in the same genetic pathway as SweDC-FtsEX-CwlO. Representative plates from one of three biological replicates are shown.
(TIF)

S11 Fig SweD-sfGFP localizes to discrete foci throughout the cell membrane. Representative image of a strain (BYB592) in which SweD-sfGFP is the sole source of SweD protein. Cells were grown to mid-exponential phase in CH medium and immobilized on 2% agarose CH pads. GFP channel (left) and phase contrast (right) are shown. Consistent with a role in regulating the elongation hydrolase CwlO, SweD-sfGFP localized in discrete foci throughout the cell membrane. The ~2-fold increased fluorescence at division sites is due to the double-membrane septum. Time-lapse imaging reveals a mixed population of diffusive and immobile foci when SweD-sfGFP is expressed at low levels. Cells expressing SweD-sfGFP are viable in the absence of LytE. The representative image is from one of three biological replicates. Scale bar indicates 2 μm.
(TIF)

S1 Text Supplementary materials and methods. (DOCX)

S1 Table *Bacillus subtilis* strains used in this study. All strains, their genotypes, and sources are listed in this table.
(PDF)

S2 Table Plasmids used in this study. All plasmids and their sources are listed in this table.
(PDF)

S3 Table Oligonucleotide primers used in this study. All oligonucleotides used for plasmid construction, gene deletion, or sequencing are listed in this table. Capital letters were used for restriction endonuclease recognition sites and underlined letters indicate mutated bases.
(PDF)
