# Supplementary figures and images for: SweC and SweD are essential co-factors of the FtsEX-CwlO cell wall hydrolase complex in Bacillus subtilis
Source: PLoS Genet. 2019 Aug 22;15(8):e1008296. doi: 10.1371/journal.pgen.1008296 (PMC6705773; doi:10.1371/journal.pgen.1008296)

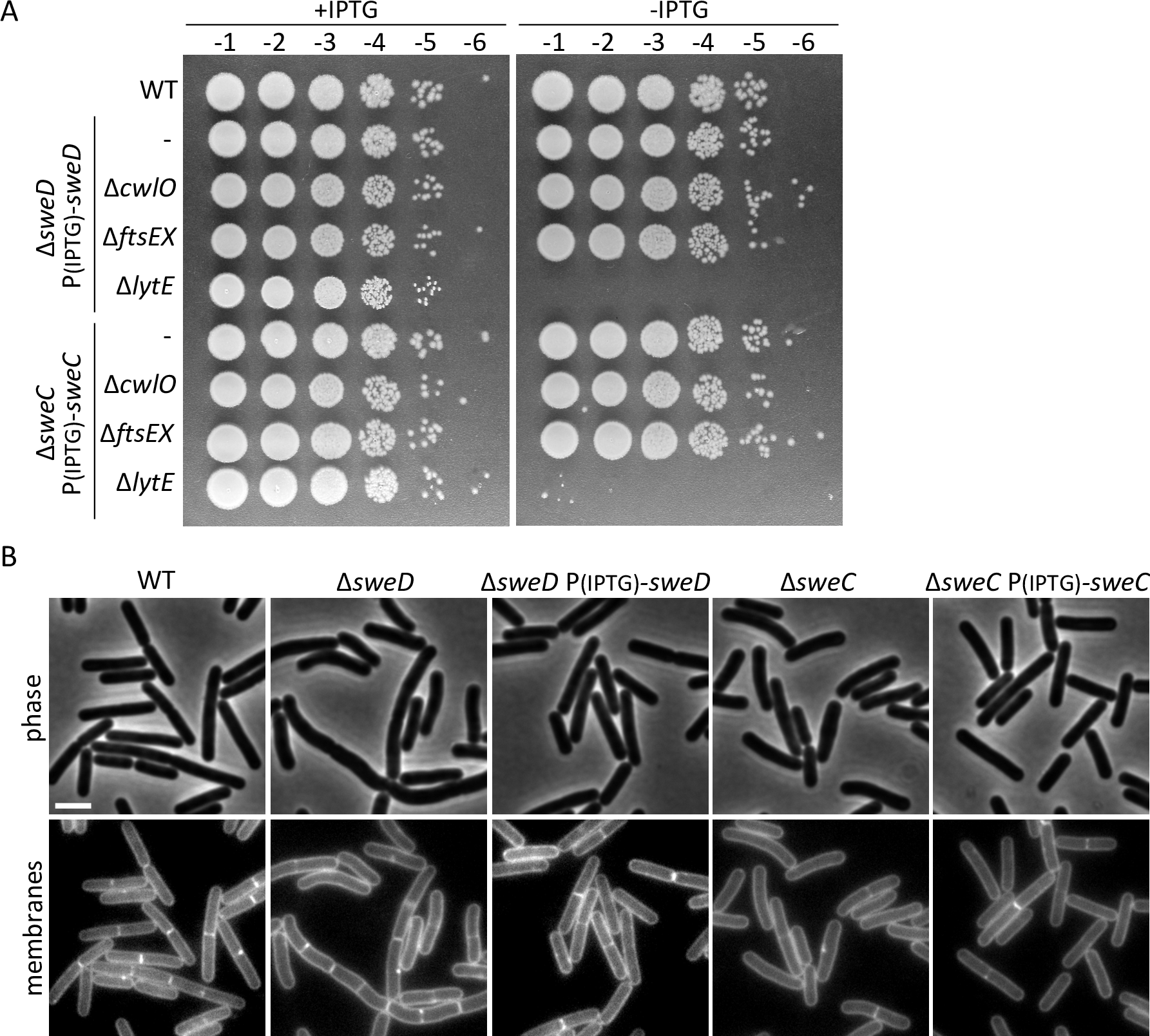

Supplement: S1 Fig — (A) sweD and sweC are synthetically lethal with lytE but not cwlO or ftsEX. Spot dilutions of the indicated strains in the presence and absence of inducer. All strains were grown in the presence of IPTG (500 μM) to an optical density of ∼2.0. The cultures were washed twice without inducer, resuspended at an OD600 of 1.5, and 10-fold serially diluted. Five microliters of each dilution were spotted onto LB agar plates with and without IPTG (500 μM). Representative plates from one of three biological replicates are shown. (B) Cells lacking SweD or SweC have similar morphological defects. Exponentially growing cells were stained with the lipophilic dye TMA-DPH and examined by fluorescence and phase-contrast microscopy. The representative images shown are from one of three independent experiments. Scale bar indicates 2 μm. (TIF) [file pgen.1008296.s001.tif]

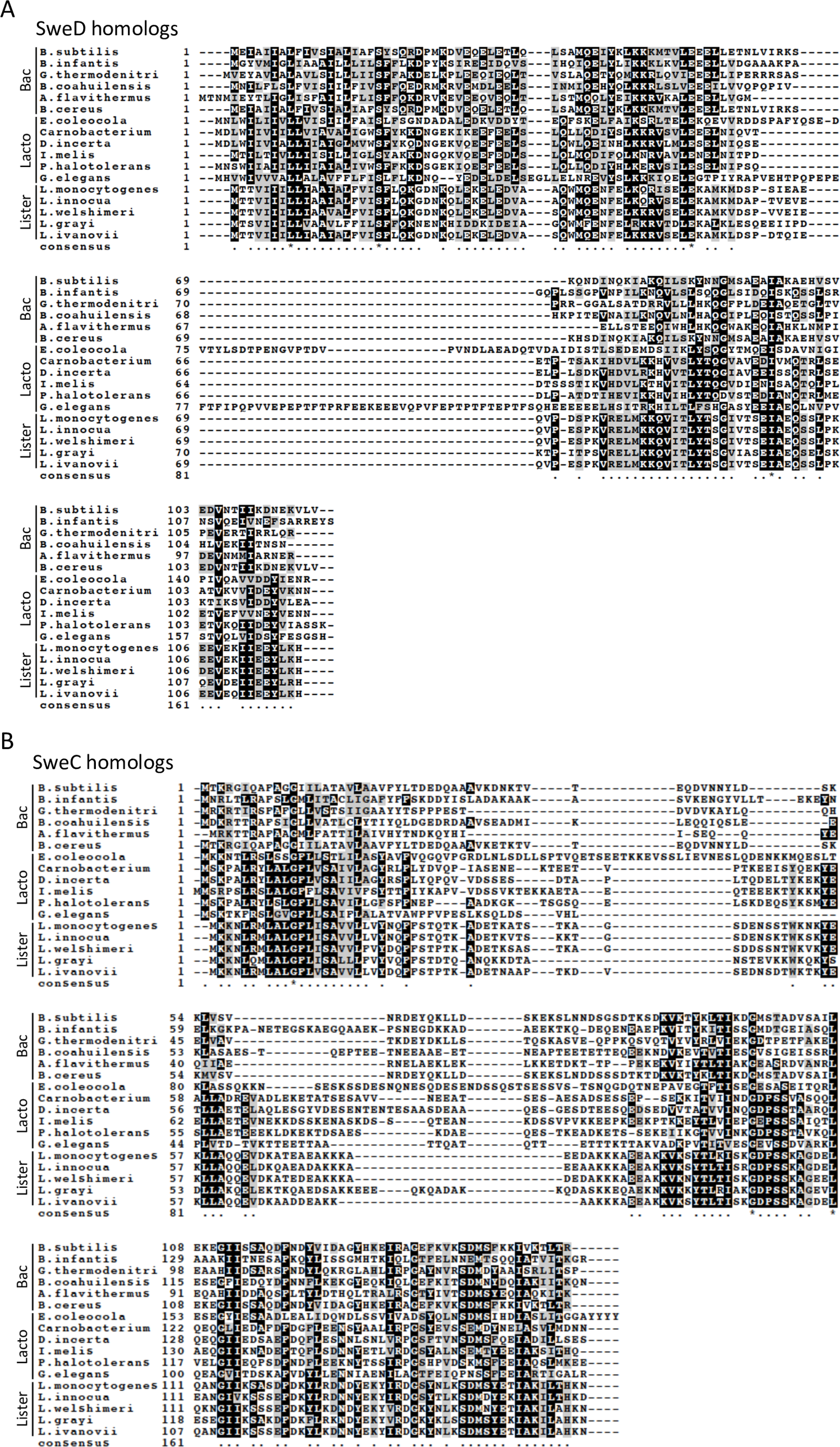

Supplement: S2 Fig — (A-B) Amino acid alignments of a subset of SweD and SweC orthologs from Bacilliaceae (Bac), Lactobacillales (Lacto), and Listeriaceae (Lister) family members. SweC orthologs were identified by PSI-BLAST and were analyzed manually for the presence of a putative N-terminal TM segment using TMHMM [42] and a C-terminal LysM-like domain using HHPred [82]. SweD orthologs were identified by analyzing the gene upstream of sweC for the presence of an N-terminal TM segment and a coiled-coil domain using COILS [83]. Alignments were generated using the Clustal Omega server [84] and shaded using BOXSHADE (https://embnet.vital-it.ch/software/BOX_form.html). Identical (black) and conserved (grey) residues are highlighted. Entrez database gene names are: yqzD (BSU24930), N288_18180, GTNG_2391, WP_010173423, Aflv_0885, CUB26036, HMPREF9257_0979, YqzD, SAMN04488506_1998, SAMN04488559_10551, SAMN04489868_11726, HMPREF0446_00721, LMO1334, LIN1371, LWE1349, HMPREF0556_12475, LIV_1285, yqzC (BSU24940), N288_18185, GTNG_2392, WP_010173421, Aflv_0884, CUB26039, HMPREF9257_0980, YqzC, SAMN04488506_1999, SAMN04488559_10550, SAMN04489868_11725, HMPREF0446_00720, LMO1333, LIN1370, LWE1348, HMPREF0556_12476, LIV_1284. (TIF) [file pgen.1008296.s002.tif]

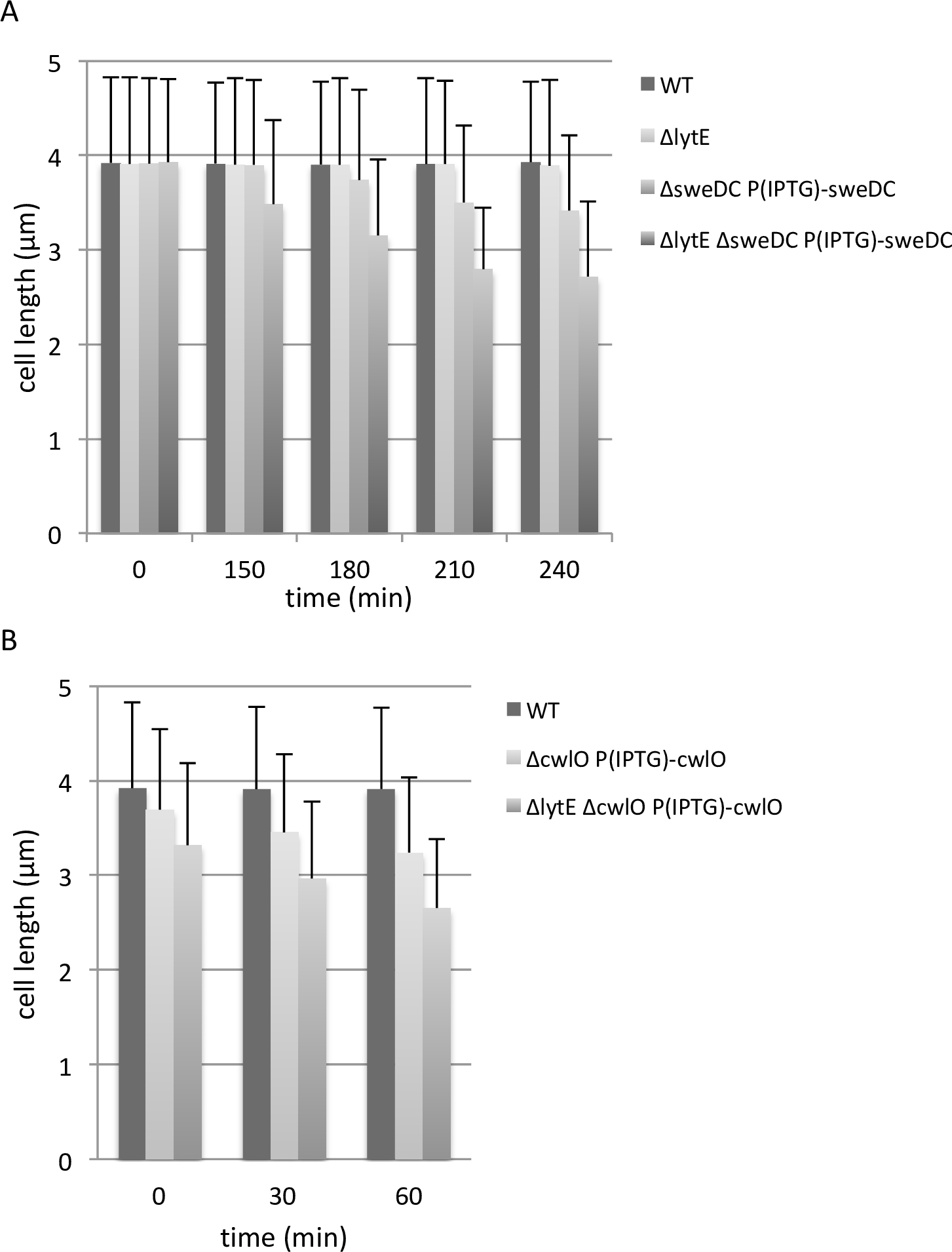

Supplement: S3 Fig — (A) mCherry images from the time-course experiment presented in Fig 2B were analyzed using Oufti [75] to assess cell length at the indicated time points after removal of IPTG. Strains were wild-type (WT) (BDR2649), ΔlytE (BYB373), ΔsweDC, P(IPTG)-sweDC (BYB360) and ΔlytE, ΔsweDC, P(IPTG)-sweDC (BYB362). >350 cells were analyzed at each time point. (B) mCherry images from the time-course experiment presented in Fig 2C were analyzed using Oufti to assess cell length at the indicated time points. Strains analyzed were wild-type (WT) (BDR2649), ΔcwlO, P(IPTG)-cwlO (BYB265) and ΔlytE, ΔcwlO, P(IPTG)-cwlO (BYB279). >350 cells were analyzed at each time point. (TIF) [file pgen.1008296.s003.tif]

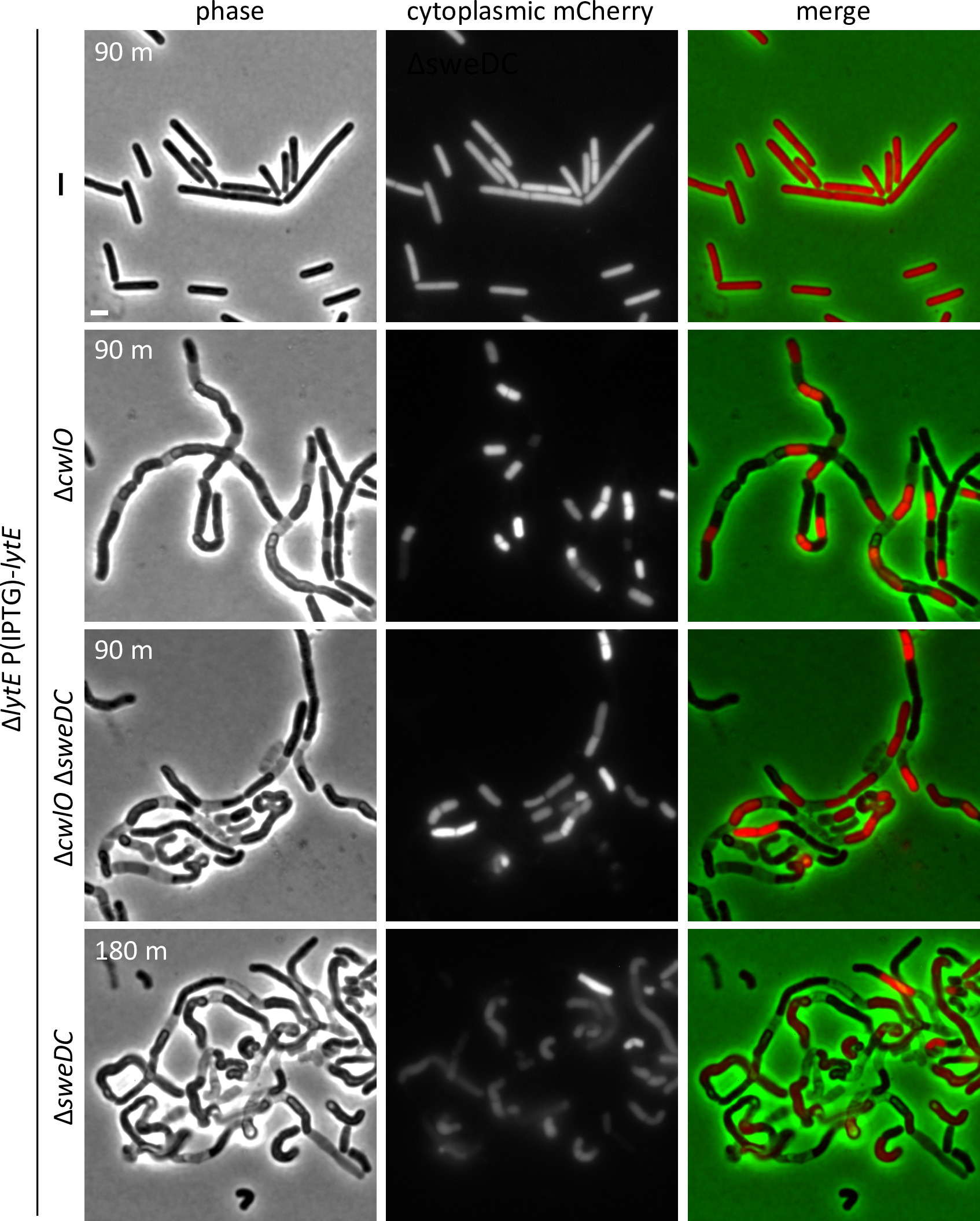

Supplement: S4 Fig — Cytological analysis comparing the terminal phenotypes of strains depleted of LytE in the absence of sweDC, cwlO, or both. The indicated strains (BYB1181, BYB1185, BYB1447, BYB1446) were grown to exponential phase in CH medium supplemented with IPTG (500 μM), washed twice with medium lacking inducer, back-diluted to an OD600 of 0.05 (BYB1181, BYB1446) or 0.1 (BYB1185, BYB1447) and grown to mid-exponential phase in the absence of inducer. Cells were examined by fluorescence microscopy every 30 min. Representative images 90 min after the removal of IPTG are shown. The ΔsweDC mutant was visualized 180 minutes after LytE shut-off because more time was required to reach the terminal phenotype. Phase contrast (phase), cytoplasmic mCherry fluorescence, and an overlay (merge) are shown. The representative images are from one of three independent experiments. Scale bar indicates 2 μm. The apparent differences in chaining of the various mutants in the images presented do not reflect cell separation defects. In larger fields of cells there was no discernable difference in cell separation among the strains presented. (TIF) [file pgen.1008296.s004.tif]

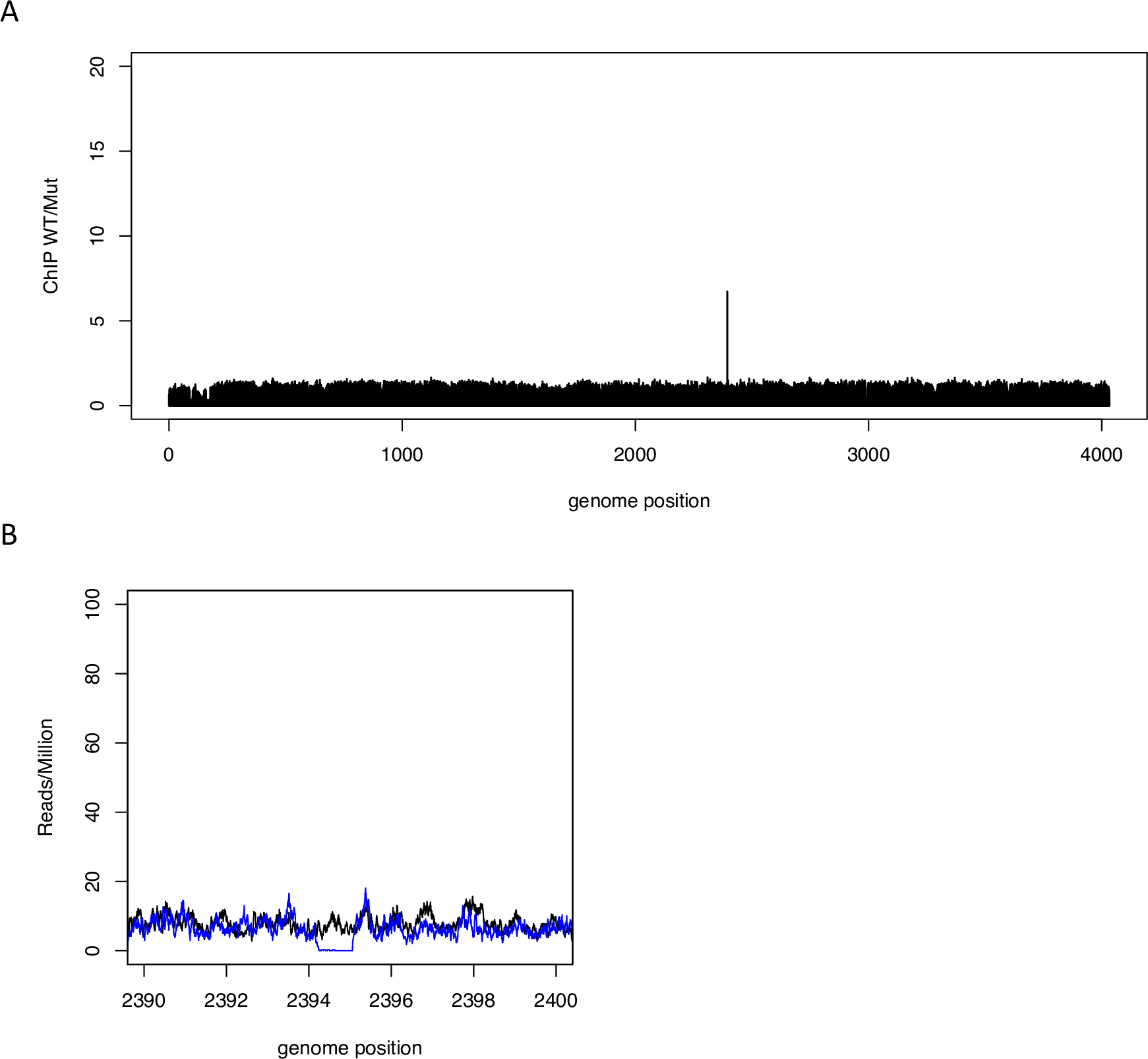

Supplement: S5 Fig — (A) The ratio of normalized ChIP-seq reads obtained in wild-type and the ΔsweDC mutant plotted across the genome. ChIP-seq using anti-SweD antibodies was performed on wild-type and ΔsweDC cells. Sequencing reads from both samples were normalized to the total number of reads for each sample. The ratio of normalized WT to ΔsweDC reads was plotted in 1 kb bins. The peak at 2394 kb overlaps the sweDC locus. (B) Zoom-in to the sweDC locus. ChIP-seq reads of wild-type (black line) and ΔsweDC mutant (blue line) were plotted at genome location 2390–2400 kb. The ChIP-seq plots of both sample show similar profiles, except at the sweDC locus explaining the high ChIP-seq ratio at this position in (A). (TIF) [file pgen.1008296.s005.tif]

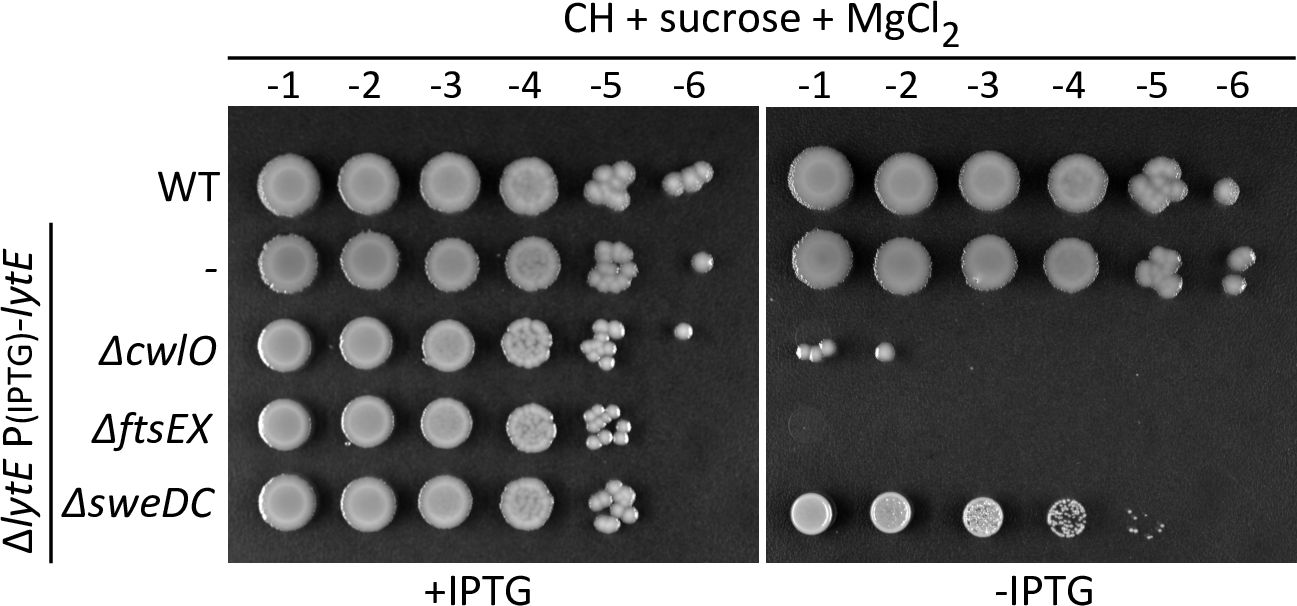

Supplement: S6 Fig — Spot dilutions of the indicated strains (PY79, BYB32, BYB35, BYB36, BYB515) in the presence and absence of inducer. All strains were grown in the presence of IPTG (500 μM) to an optical density of ∼2.0. The cultures were washed twice without inducer, resuspended at an OD600 of 1.5, and 10-fold serially diluted. Five microliters of each dilution was spotted onto CH plates supplemented with 20 mM MgCl2 and 0.25 M sucrose with and without inducer. Representative plates from one of three biological replicates are shown. (TIF) [file pgen.1008296.s006.tif]

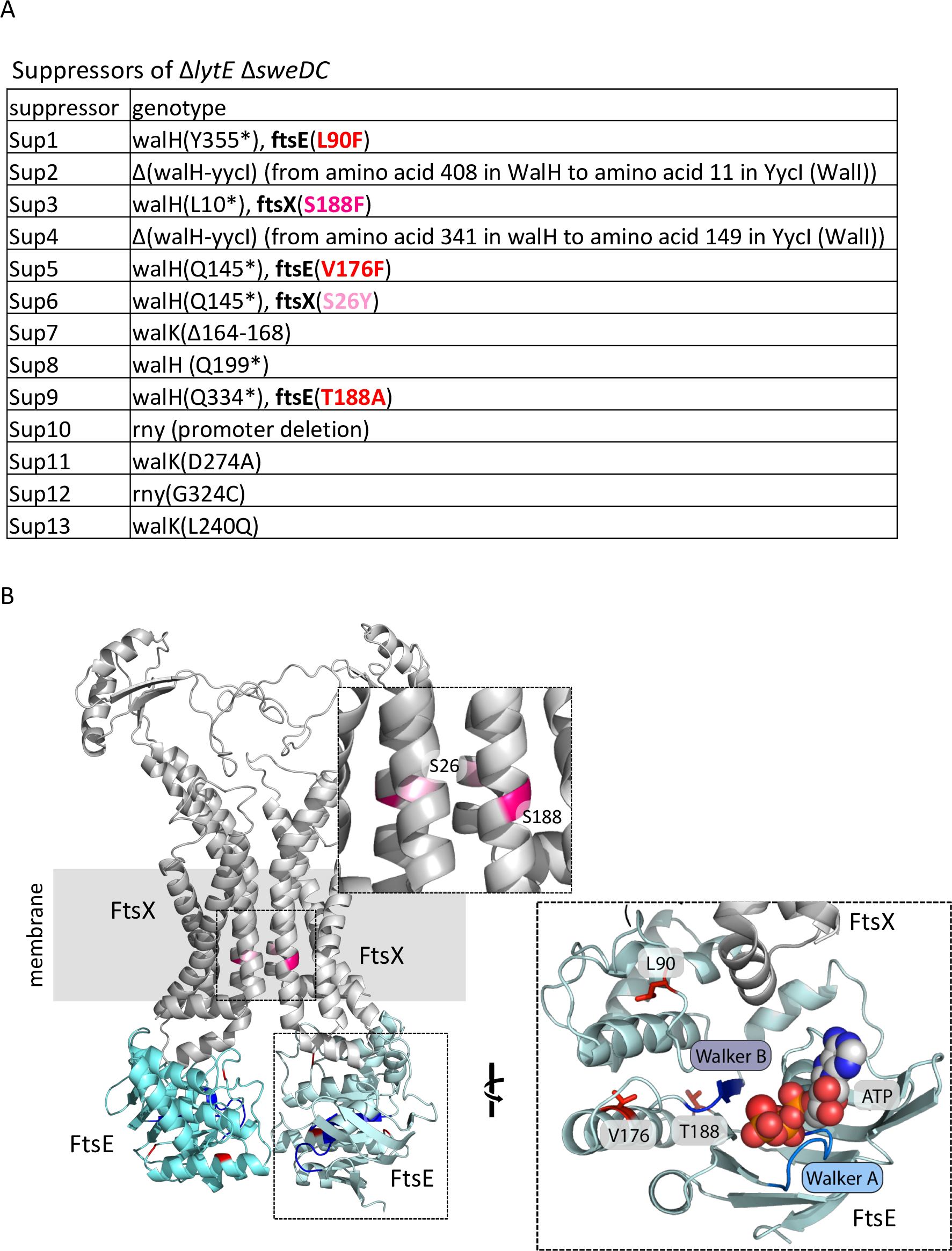

Supplement: S7 Fig — (A) Mutations in the ΔsweDC ΔlytE suppressor strains identified by whole genome re-sequencing. Most suppressors had loss-of-function mutations in walH encoding a negative regulator of the WalK sensor kinase [45]. Five of these had second-site mutations in ftsE or ftsX. All five mutations in ftsE or ftsX were separately reconstructed and found to suppress the lethality of the ΔsweDC ΔlytE double mutant on defined (CH) rich medium in the presence of wild-type walH. Three suppressors had missense mutations in walK that are predicted to cause constitutive signaling [50, 85]. Two suppressors had mutations in rny encoding Rnase Y. The cwlO mRNA was shown to be stabilized in the absence of RNAse Y [86]. However, ~2-fold over-expression of CwlO was not sufficient to suppress the ΔsweDC ΔlytE mutant. (B) Homology model of B. subtilis FtsEX generated using the SWISS-MODEL server [87]. The structure of the Aggregatibacter actinomycetemcomitans ABC transporter MacB (PDB: 5LIL) [28] and that of the large extracellular loop of Mycobacteria tuberculosis FtsX (PDB: 4N8O) [23] were used as templates. The residues in FtsX (S26 and S188) and in FtsE (L90, V176, and T188) that were identified in the suppressor selection are highlighted in pink and red, respectively. Boxed regions highlight the TM segments in FtsX that contain the two serine residues (pink) that suppressed when substituted (top) and an FtsE monomer with ATP modeled into the nucleotide binding pocket (bottom). Walker A and B motifs are shown in light and dark blue. The three residues in FtsE (red) that suppressed when substituted are indicated. (TIF) [file pgen.1008296.s007.tif]

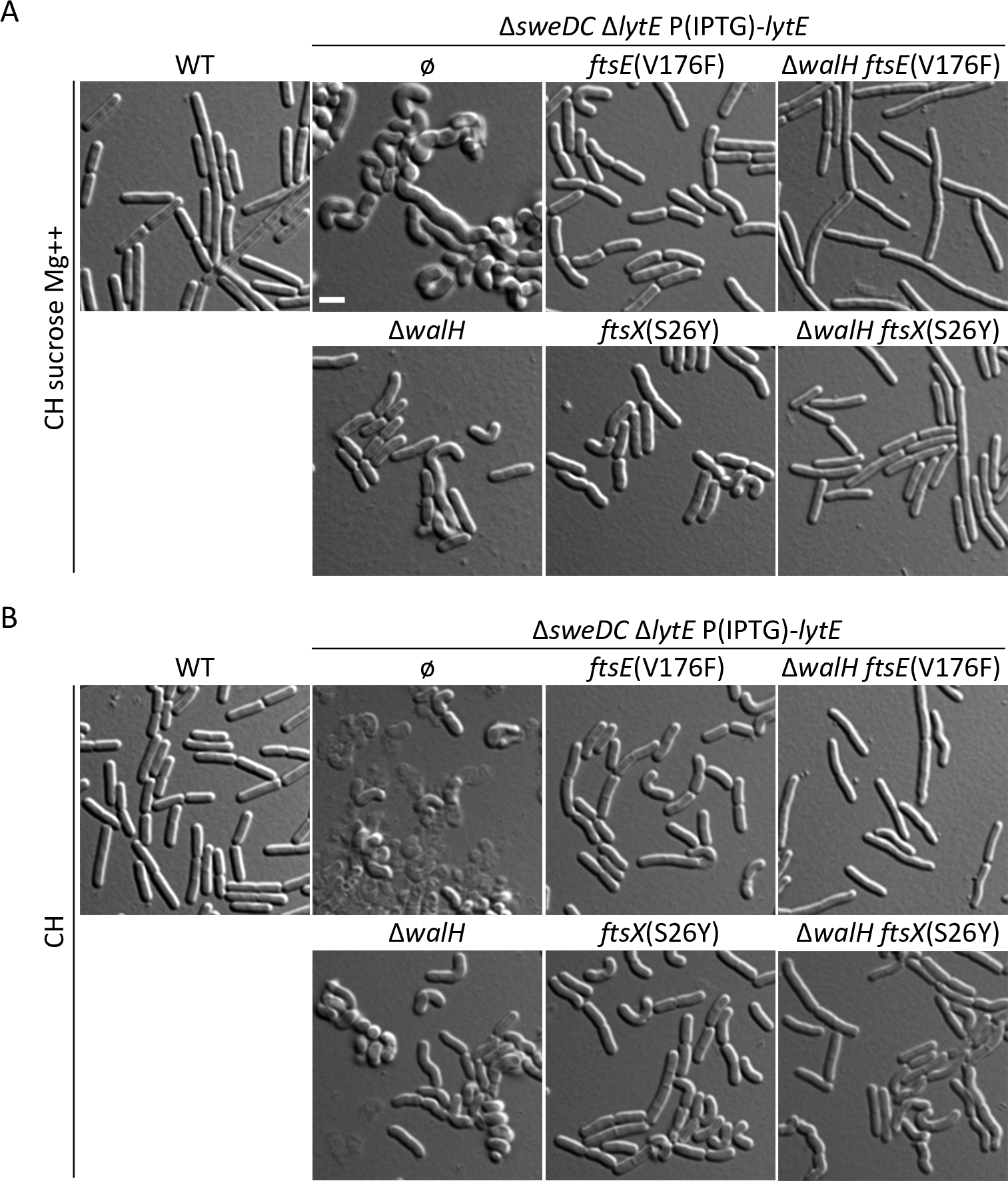

Supplement: S8 Fig — Cytological analysis of the suppressors strains under permissive (A) and restrictive conditions (B). The indicated strains were grown to exponential phase in CH medium in the presence of IPTG (500 μM). The cultures were washed twice with medium lacking inducer, back-diluted to an OD600 of 0.05 in permissive or restrictive culture medium (CH medium supplemented with 20 mM MgCl2 and 0.25 M sucrose and CH medium alone, respectively). Cells were immobilized on 2% agarose CH (20 mM MgCl2 and 0.25 M Sucrose) or agarose CH pads and observed using differential interference contrast (DIC) microscopy. Representative images from one of three biological replicates are shown. Scale bar indicates 2 μm. (TIF) [file pgen.1008296.s008.tif]

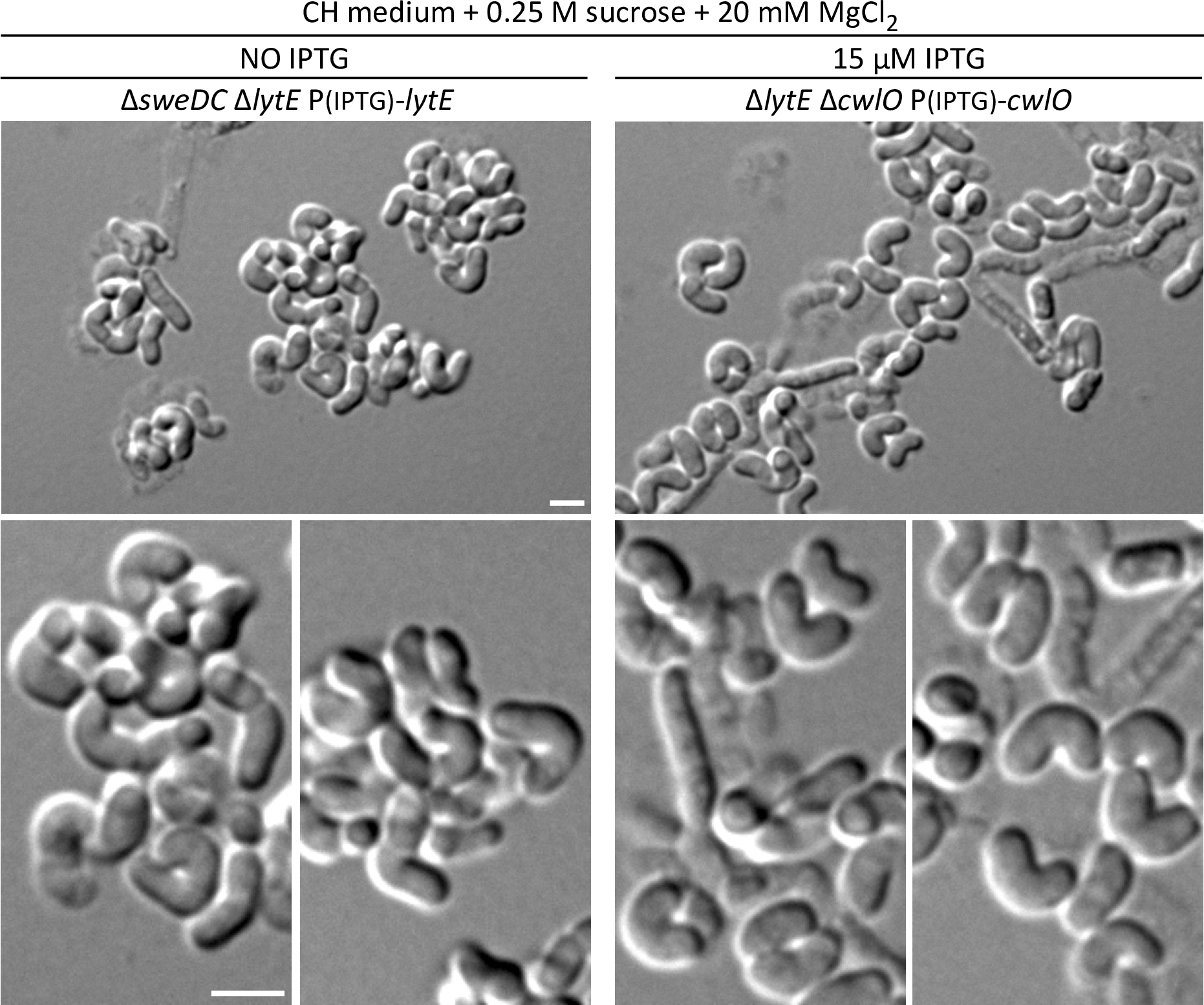

Supplement: S9 Fig — Comparison of cell morphologies in strains lacking SweDC and depleted of LytE (BYB515) or expressing low levels of CwlO in the absence of LytE (BYB287). Both strains were grown under permissive conditions (CH medium supplemented with 0.25 M sucrose and 20 mM MgCl2). Representative DIC images are shown. Cells were grown to exponential phase in CH medium supplemented with IPTG (500 μM), washed twice with medium lacking inducer, back-diluted to an OD600 of 0.02 under permissive growth conditions in the presence (BYB287) or absence (BYB515) of 15 μM IPTG. After five generations, cells were immobilized on 2% agarose, 20 mM MgCl2 and 0.25 M sucrose CH pads and observed using differential interference contrast (DIC) microscopy. The representative images shown are from one of three biological replicates. Scale bar indicates 2 μm. (TIF) [file pgen.1008296.s009.tif]

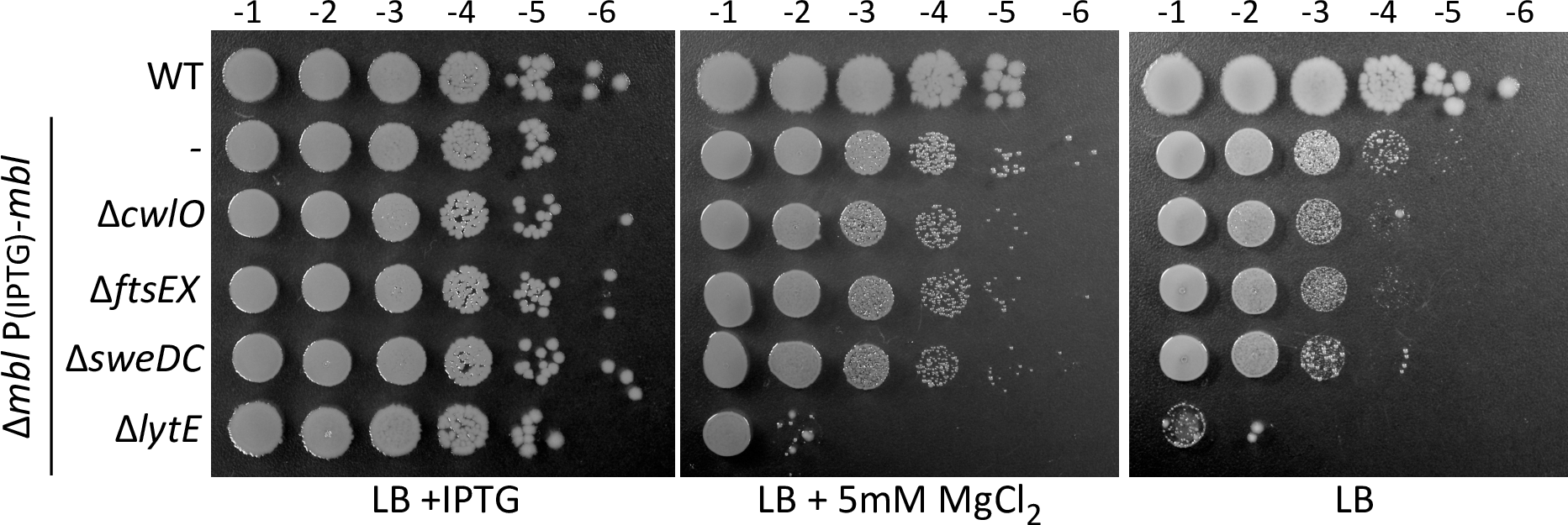

Supplement: S10 Fig — Spot dilutions of the indicated strains in the presence and absence of inducer. All strains were grown in the presence of IPTG (500 μM) to an optical density of ∼2.0. The cultures were washed twice without inducer, resuspended at an OD600 of 1.5, and 10-fold serially diluted. Five microliters of each dilution were spotted onto LB agar plates with and without IPTG (500 μM) and LB agar plates supplemented with 5 mM MgCl2, a semi-permissive condition for cells lacking Mbl. Cells depleted of Mbl on LB agar in the presence or absence of 5 mM MgCl2 have a slow-growth phenotype. The growth defect is modestly enhanced to a similar degree in the absence of CwlO, FtsEX, or SweDC. However, in the absence of LytE, depletion of Mbl results in 4-log plating defect as reported previously [22]. These data provide evidence that Mbl is in the same genetic pathway as SweDC-FtsEX-CwlO. Representative plates from one of three biological replicates are shown. (TIF) [file pgen.1008296.s010.tif]

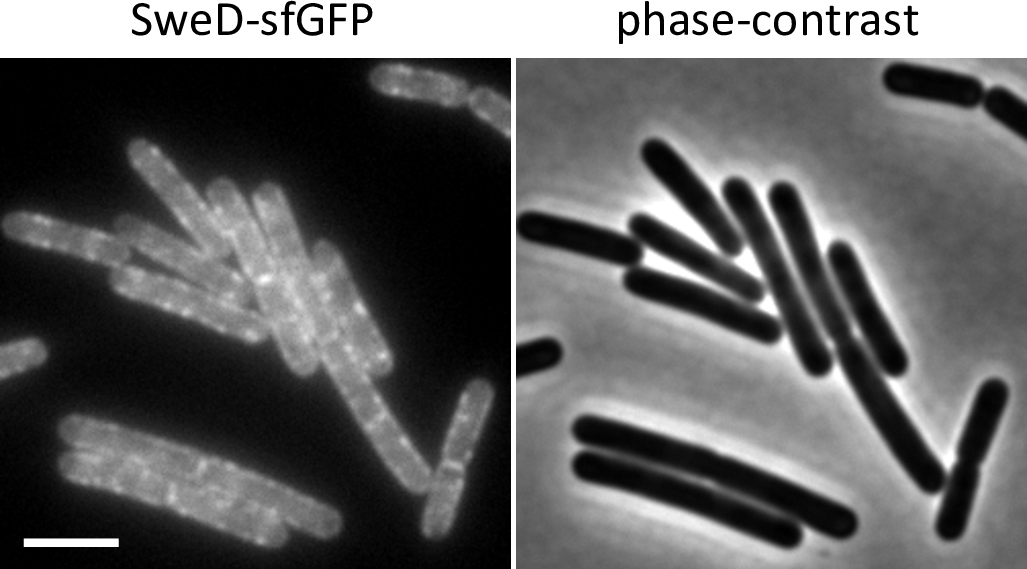

Supplement: S11 Fig — Representative image of a strain (BYB592) in which SweD-sfGFP is the sole source of SweD protein. Cells were grown to mid-exponential phase in CH medium and immobilized on 2% agarose CH pads. GFP channel (left) and phase contrast (right) are shown. Consistent with a role in regulating the elongation hydrolase CwlO, SweD-sfGFP localized in discrete foci throughout the cell membrane. The ~2-fold increased fluorescence at division sites is due to the double-membrane septum. Time-lapse imaging reveals a mixed population of diffusive and immobile foci when SweD-sfGFP is expressed at low levels. Cells expressing SweD-sfGFP are viable in the absence of LytE. The representative image is from one of three biological replicates. Scale bar indicates 2 μm. (TIF) [file pgen.1008296.s011.tif]
