# Supplementary material for: SweC and SweD are essential co-factors of the FtsEX-CwlO cell wall hydrolase complex in Bacillus subtilis
Source: PLoS Genet. 2019 Aug 22;15(8):e1008296. doi: 10.1371/journal.pgen.1008296 (PMC6705773; doi:10.1371/journal.pgen.1008296)
Supplement: S1 Text — (DOCX) [file pgen.1008296.s012.docx]

**SUPPLEMENTARY MATERIALS AND METHODS**

**Plasmid construction.**

All plasmid inserts were confirmed by Sanger sequencing.

**pYB02 [ycgO::Pspank-sweDC (erm)]** was generated by ligation with a SpeI-HindIII PCR product containing *sweDC* (amplified with oligonucleotide primers oYB38 and oYB33 from PY79 genomic DNA) and pER065 [ycgO::Pspank (*erm*)].

**pYB04 [His6-SUMO-sweC (soluble domain) (amp)]** was generated by ligation with a BamHI-XhoI PCR product encoding the SweC soluble domain (amplified with oligonucleotide primers oYB41 and oYB42 from PY79 genomic DNA) and pTD68 [His6-SUMO (amp)] [5].

**pYB05 [His6-SUMO-sweD (soluble domain) (amp)]** was generated by ligation with a BamHI-XhoI PCR product encoding the SweD soluble domain (amplified with oligonucleotide primers oYB43 and oYB44 from PY79 genomic DNA) and pTD68 [His6-SUMO (amp)].

**pYB16 [amyE::sweD-sfGFP (kan)]** was generated in a 3-way isothermal assembly reaction [8] with two PCR products containing *sweD* and super-folder GFP (*sfGFP*) (amplified with oligonucleotide primers oYB61 and oYB62, and oYB63 and oYB64 from PY79 genomic DNA and pDHL502 [9] respectively) and pER82 [amyE::kan] cut with BamHI and EcoRI.

**pYB20 [ycgO::Pspank-sweD (spec)]** was generated by ligation with a SpeI-HindIII PCR product containing SweD (amplified with oligonucleotide primers oYB38 and oYB67 from PY79 genomic DNA) and pER107 [ycgO::Pspank (spec)].

**pYB21 [ycgO::Pspank-sweC (spec)]** was generated by ligation with a SpeI-HindIII PCR product containing SweC (amplified with oligonucleotide primers oYB33 and oYB68 from PY79 genomic DNA) and pER107 [ycgO::Pspank (spec)].

**pYB61 [yvbJ::PxylA-lytE (cat)]** was generated by restriction-free cloning [10]. A *lytE* PCR product (amplified with oligonucleotide primers oYB162 and oYB163 from PY79 genomic DNA) which contained 5’ extensions that annealed to the destination vector pMS039 [yvbJ::PxylA (cat)] was used as oligonucleotides for a second PCR using pMS039 as template.

**pYB130 [ycgO::Pspank-sweDC ∆25-57 (spec)]** was generated in a 3-way isothermal assembly reaction [8] with two PCR products containing *sweDC*∆25-57 (amplified with oligonucleotide primers oYB313 and oYB315, and oYB316 and oYB314 from PY79 genomic DNA) and pER107 [ycgO::Pspank (spec)] cut with SpeI and HindIII.

**pYB132 [ycgO::Pspank-sweDC ∆74-108 (spec)]** was generated in a 3-way isothermal assembly reaction [8] with two PCR products containing *sweDC* ∆74-108 (amplified with oligonucleotide primers oYB313 and oYB319, and oYB320 and oYB314 from PY79 genomic DNA) and pER107 [ycgO::Pspank (spec)] cut with SpeI and HindIII.

**pYB134 [ycgO::Pspank-sweDC D104A-V105A (spec)]** was generated in a 3-way isothermal assembly reaction [8] with two PCR products containing *sweDC* D104A-V105A (amplified with oligonucleotide primers oYB313 and oYB323, and oYB324 and oYB314 from PY79 genomic DNA) and pER107 [ycgO::Pspank (spec)] cut with SpeI and HindIII.

**pYB135 [ycgO::Pspank-sweDC ∆LysM (spec)]** was generated in a 2-way isothermal assembly reaction [8] with a PCR product containing *sweDC* ∆LysM (amplified with oligonucleotide primers oYB313 and oYB325 from PY79 genomic DNA) and pER107 [ycgO::Pspank (spec)] cut with SpeI and HindIII.

**pYB24, pYB26, pYB29, pYB30, pYB42, pYB44, pYB36, pYB37, pYB39, pYB147, pYB183** used in the BACTH assay were generated by restriction-free cloning [10]. PCR products, which contain 5’ extensions that anneal to the destination vectors pEB354 [pUT25 (kan)] or pEB355 [pUT18 (amp)] were used as oligonucleotides for a second PCR using pEB354 or pEB355 as template. PCR products were generated with oYB91 and oYB93 (pYB24), oYB92 and oYB93 (pYB26), oYB100 and oYB102 (pYB29), oYB98 and oYB99 (pYB30), oYB97 and oYB93 (pYB42), oYB95 and oYB102 (pYB44), oYB109 and oYB111 (pYB36), oYB110 and oYB111 (pYB37), oYB113 and oYB114 (pYB39), oYB134 and oYB111 (pYB183) using PY79 genomic DNA as template, and oYB110 and oYB111 (pYB147) using pYB130 as template.

**Strain construction.**

Gene deletion mutants were each generated by isothermal assembly [8] and direct transformation in *B.* *subtilis*. Each isothermal assembly reaction contained three PCR products: an antibiotic resistance cassette and upstream and downstream regions (~1.2 kb each) that flank the gene of interest. The kanamycin, tetracycline and erythromycin resistance cassettes, flanked by lox66 and lox71 sites, were amplified by PCR with oJM28 and oJM29 [2] from pWX470, pWX469 and pWX467, respectively [2, 3]. Upstream and downstream regions were amplified from PY79 genomic DNA to generate: *sweD::kan* (oligonucleotide primers oYB34, oYB35, oYB141, and oYB142), *sweC::kan* (oligonucleotide primers oYB34, oYB143, oYB36, and oYB37), *sweDC::kan and sweDC::erm* (oligonucleotide primers oYB34, oYB35, oYB36, and oYB37), walH::tet (oligonucleotide primers oYB127, oYB128, oYB129, and oYB130).

**BYB21 [*ycgO*::Pspank-*lytE* (*spec*)]** was generated by transformation of BKM400 (*ycgO*::*cat*) with an isothermal assembly reaction containing a *lytE* PCR product (amplified with oligonucleotide primers oJM103 and oJM104 [2] from PY79 genomic DNA) and pER107 [*ycgO*::Pspank (*spec*)] cut with SpeI and SphI.

**BYB461 [*ftsEX* (*erm*)]** was generated by transformation of PY79 with an isothermal assembly reaction containing the Erythromycin (Erm) resistance cassette and upstream and downstream regions. The Erm cassette was amplified by PCR with oJM28 and oJM29 [2] from pW467 [3] and the upstream and downstream regions were amplified with oligonucleotide primers oYB83 and oYB84, and oYB85 and oYB86 from PY79 genomic DNA.

BYB522 [*ftsE*(V176F) (*erm*)] was generated by transformation of BJM68 [*ftsEX::kan*] [2] with an isothermal assembly reaction containing two PCR products that contained *ftsE*(V176F)-*ftsX* *erm* (amplified with oligonucleotide primers oJM54 and oYB370, and oYB371 and oYB86 from BYB461 [*ftsEX* (*erm*)] genomic DNA).

BYB523 [*ftsX*(S26Y) (*erm*)] was generated by transformation of BJM68 [*ftsEX::kan*] [2] with an isothermal assembly reaction containing two PCR products that contained *ftsE-ftsX*(S26Y) *erm* (amplified with oligonucleotide primers oJM54 and oYB372, and oYB373 and oYB86 from BYB461 [*ftsEX* (*erm*)] genomic DNA).

BYB366, BYB367 and BYB368 [*sweDC::kan, lytE::cat*] were generated by transformation of BYB339 [*sweDC::kan*] with genomic DNA isolated from BJM76 [*lytE::cat*] [2]. Transformants were selected and maintained on CH agar plates supplemented with MgCl_2_ (20 mM), Sucrose (0.25M), and chloramphenicol (5 µg/mL).

**Antibiotic cassette loop-out**

Strains with *loxP*-flanked antibiotic cassettes were transformed with pDR244 [3], a temperature-sensitive plasmid with constitutively expressed Cre recombinase marked with a *spec* resistance gene. Transformants were selected on LB supplemented with 100 μg/ml Spectinomycin at 30 °C, a permissive temperature for pDR244 replication. Transformants were then streaked on LB agar plates and incubated at 42°C, a restrictive temperature for plasmid replication. Single colonies were then re-streaked on LB, LB(Spec), and LB(Kan) and incubated at 37 °C. Strains that grew on LB, but not LB(Spec) or LB(Kan), had lost pDR244 and the kanamycin resistance cassette. Markerless deletions were confirmed by PCR with oligonucleotide primers flanking the deletion.

**REFERENCES**

1. Youngman PJ, Perkins JB, Losick R. Genetic transposition and insertional mutagenesis in Bacillus subtilis with Streptococcus faecalis transposon Tn917. Proc Natl Acad Sci U S A. 1983 Apr;80(8):2305-9. PubMed PMID: 6300908; PubMed Central PMCID: PMC393808.

2. Meisner J, Montero Llopis P, Sham LT, Garner E, Bernhardt TG, Rudner DZ. FtsEX is required for CwlO peptidoglycan hydrolase activity during cell wall elongation in Bacillus subtilis. Mol Microbiol. 2013;89(6):1069-83. doi: 10.1111/mmi.12330. PubMed PMID: 23855774; PubMed Central PMCID: PMCPMC3786131.

3. Wang X, Tang OW, Riley EP, Rudner DZ. The SMC condensin complex is required for origin segregation in Bacillus subtilis. Curr Biol. 2014;24(3):287-92. doi: 10.1016/j.cub.2013.11.050. PubMed PMID: 24440393; PubMed Central PMCID: PMCPMC3947903.

4. Meeske AJ, Sham LT, Kimsey H, Koo BM, Gross CA, Bernhardt TG, et al. MurJ and a novel lipid II flippase are required for cell wall biogenesis in Bacillus subtilis. Proc Natl Acad Sci U S A. 2015;112(20):6437-42. doi: 10.1073/pnas.1504967112. PubMed PMID: 25918422; PubMed Central PMCID: PMCPMC4443310.

5. Morlot C, Uehara T, Marquis KA, Bernhardt TG, Rudner DZ. A highly coordinated cell wall degradation machine governs spore morphogenesis in Bacillus subtilis. Genes Dev. 2010 Feb 15;24(4):411-22. doi: 10.1101/gad.1878110. PubMed PMID: 20159959; PubMed Central PMCID: PMC2816739.

6. Battesti A, Bouveret E. Improvement of bacterial two-hybrid vectors for detection of fusion proteins and transfer to pBAD-tandem affinity purification, calmodulin binding peptide, or 6-histidine tag vectors. Proteomics. 2008 Nov;8(22):4768-71. doi: 10.1002/pmic.200800270. PubMed PMID: 18924111.

7. Gully D, Bouveret E. A protein network for phospholipid synthesis uncovered by a variant of the tandem affinity purification method in Escherichia coli. Proteomics. 2006 Jan;6(1):282-93. PubMed PMID: 16294310.

8. Gibson DG. Enzymatic assembly of overlapping DNA fragments. Methods Enzymol. 2011;498:349-61. doi: 10.1016/B978-0-12-385120-8.00015-2. PubMed PMID: 21601685.

9. Landgraf D, Okumus B, Chien P, Baker TA, Paulsson J. Segregation of molecules at cell division reveals native protein localization. Nat Methods. 2012 Apr 8;9(5):480-2. doi: 10.1038/nmeth.1955. PubMed PMID: 22484850; PubMed Central PMCID: PMC3779060.

10. van den Ent F, Löwe J. RF cloning: a restriction-free method for inserting target genes into plasmids. J Biochem Biophys Methods. 2006 Apr 30;67(1):67-74. PubMed PMID: 16480772.
