# Supplementary material for: SweC and SweD are essential co-factors of the FtsEX-CwlO cell wall hydrolase complex in Bacillus subtilis
Source: PLoS Genet. 2019 Aug 22;15(8):e1008296. doi: 10.1371/journal.pgen.1008296 (PMC6705773; doi:10.1371/journal.pgen.1008296)
Supplement: S1 Table — All strains, their genotypes, and sources are listed in this table. (PDF) [file pgen.1008296.s013.pdf]

SUPPLEMENTARY MATERIALS AND METHODS

TABLE S1: *Bacillus subtilis* strains used in this study

Strain	Genotype	Source	Figure
PY79	wild-type	Youngman et al., 1983	1, 3, 7, S1, S4, S5, S7
BDR2649	*sacA::Pveg-mCherry (spec)*	Meisner *et al.*, 2013	Fig. 2, S3
BDR776	*amyE::PxylA-gfp-spoIVFA (cat)*	This study	Fig. 3
BJM054	*cwlO::cat*	Meisner *et al.*, 2013	Fig. 4, S4
BJM072	*ftsX::kan*	Meisner *et al.*, 2013	Fig. 7
BJM076	*lytE::cat*	Meisner *et al.*, 2013	Fig. 1
BJM182	*lytE::tet, ycgO::Pspank-lytE (erm)*	Meisner *et al.*, 2013	Fig. 1
BJM196	*lytE::tet, ycgO::Pspank-lytE (erm), cwlO::kan*	Meisner *et al.*, 2013	Fig. 1
BJM272	*ftsEX::cat*	Meisner *et al.*, 2013	Fig. 4, S4
BYB021	*ycgO::Pspank-lytE (spec)*	This study	
BYB032	*lytE::cat, ycgO::Pspank-lytE (spec)*	This study	Fig. 6, S5
BYB035	*lytE::cat, ycgO::Pspank-lytE (spec), cwlO::kan*	This study	Fig. S5
BYB036	*lytE::cat, ycgO::Pspank-lytE (spec), ftsEX::kan*	This study	Fig. S5
BYB265	*cwlO::cat, ycgO::Pspank-5'UTR-cwlO (spec), sacA::Pveg-mCherry (tet)*	This study	Fig. S3
BYB279	*cwlO::cat, ycgO::Pspank-5'UTR-cwlO (spec), sacA::Pveg-mCherry (tet), lytE::erm*	This study	Fig. 2, S3
BYB287	*cwlO::cat, ycgO::Pspank-5'UTR-cwlO (spec), sacA::Pveg-mCherry (tet), lytE::kan*	This study	Fig. S7
BYB304	*ΔsweC::lox72*	This study	Fig. 4, S1
BYB325	*ΔsweC::lox72, ycgO::Pspank-lytE (erm), lytE::cat*	This study	Fig. 1
BYB329	*ΔsweD::lox72, ycgO::Pspank-lytE (erm), lytE::cat*	This study	Fig. 1
BYB339	*sweDC::kan*	This study	Fig. 4, 6
BYB356	*ycgO::Pspank-sweDC (erm), sweDC::kan*	This study	Fig. 1
BYB358	*ycgO::Pspank-sweDC (erm), sweDC::kan, lytE::cat*	This study	Fig. 1
BYB360	*ycgO::Pspank-sweDC (erm), sweDC:kan, sacA::Pveg-mCherry (tet)*	This study	Fig. S3
BYB362	*ycgO::Pspank-sweDC (erm), sweDC:kan, lytE::cat, sacA::Pveg-mCherry (tet)*	This study	Fig. 2, S3
BYB366	*sweDC::kan, lytE::cat clone#1*	This study	
BYB367	*sweDC::kan, lytE::cat clone#2*	This study	
BYB368	*sweDC::kan, lytE::cat clone#3*	This study	
BYB370	*sacA::Pveg-mCherry (spec), sweDC::kan*	This study	Fig. 2
BYB371	*sacA::Pveg-mCherry (spec), cwlO::cat*	This study	Fig. 2
BYB372	*sacA::Pveg-mCherry (spec), ftsEX::cat*	This study	Fig. 2
BYB373	*sacA::Pveg-mCherry (spec), lytE::cat*	This study	Fig. 2
BYB374	*sacA::Pveg-mCherry (spec), sweDC::kan, cwlO::cat*	This study	Fig. 2
BYB377	*ycgO::Pspank-sweDC (erm), sweDC::kan, cwlO::cat*	This study	Fig. 1
BYB405	*ftsEX::kan, amyE::ftsE(K41A)-ftsX (spec)*	This study	Fig. S4
BYB439	*ftsEX::kan, ycgO::Pspank-ftsEX (erm), lytE::cat, sacA::Pveg-mCherry (tet)*	This study	Fig. 2
BYB461	*ftsEX (erm)*	This study	
BYB493	*ΔsweC::lox72, ycgO::Pspank-sweC (spec)*	This study	Fig. 4, S1
BYB515	*lytE::cat, ycgO::Pspank-lytE (spec), sweDC::kan*	This study	Fig. 6, S5, S7, S8
BYB522	*ftsE(V176F) (erm)*	This study	
BYB523	*ftsX(S26Y) (erm)*	This study	
BYB529	*lytE::cat, ycgO::Pspank-lytE (spec), sweDC::kan, ftsE(V176F) (erm)*	This study	Fig. 6, S7

BYB530	*lytE::cat, ycgO::Pspank-lytE (spec), sweDC::kan, ftsX(S26Y) (erm)*	This study	Fig. 6, S7
BYB533	*lytE::cat, ycgO::Pspank-lytE (spec), sweDC::kan, walH::tet*	This study	Fig. 6, S7
BYB546	Δ*sweD::lox72*	This study	Fig. 4, S1
BYB550	Δ*sweD::lox72, ycgO::Pspank-sweD (spec)*	This study	Fig. 4, S1
BYB552	Δ*sweD::lox72, ycgO::Pspank-sweD (spec), lytE::cat*	This study	Fig. S1
BYB558	Δ*sweC::lox72, ycgO::Pspank-sweC (spec), lytE::cat*	This study	Fig. S1
BYB563	Δ*sweD::lox72, ycgO::Pspank-sweD (spec), cwlO::cat*	This study	Fig. S2
BYB564	Δ*sweD::lox72, ycgO::Pspank-sweD (spec), ftsEX::kan*	This study	Fig. S3
BYB565	Δ*sweC::lox72, ycgO::Pspank-sweC (spec), cwlO::cat*	This study	Fig. S4
BYB566	Δ*sweC::lox72, ycgO::Pspank-sweC (spec), ftsEX::kan*	This study	Fig. S5
BYB583	*ftsEX::kan, amyE::ftsE(H195Y)-ftsX (spec)*	This study	Fig. S4
BYB584	*ftsEX::kan, amyE::ftsE(D162A)-ftsX (spec)*	This study	Fig. S4
BYB585	*lytE::cat, ycgO::Pspank-lytE (spec), sweDC::kan, ftsE(V176F) (erm), walH::tet*	This study	Fig. 6, S7
BYB587	*lytE::cat, ycgO::Pspank-lytE (spec), sweDC::kan, ftsX(S26Y) (erm), walH::tet*	This study	Fig. 6, S7
BYB592	Δ*sweD::lox72 amyE::sweD-sfGFP (kan)*	This study	Fig. S9
BYB660	Δ*lytE::lox72, yvbJ::PxylA-lytE (cat), sweDC::kan*	This study	Fig. 5
BYB1181	*sacA::Pveg-mcherry (tet), lytE::cat, yvbJ::Pspank-lytE (spec)*	This study	Fig. S3
BYB1185	*sacA::Pveg-mcherry (tet), lytE::cat, yvbJ::Pspank-lytE (spec), cwlO::kan*	This study	Fig. S3
BYB1225	Δ*lytE::lox72, yvbJ::PxylA-lytE (cat), sweDC::kan, ycgO::Pspank-sweDC (erm)*	This study	Fig. 5
BYB1226	Δ*lytE::lox72, yvbJ::PxylA-lytE (cat), sweDC::kan, ycgO::Pspank-sweDC(Δ25-57) (spec)*	This study	Fig. 5
BYB1228	Δ*lytE::lox72, yvbJ::PxylA-lytE (cat), sweDC::kan, ycgO::Pspank-sweDC(Δ74-108) (spec)*	This study	Fig. 5
BYB1229	Δ*lytE::lox72, yvbJ::PxylA-lytE (cat) sweDC::kan, ycgO::Pspank-sweDC(D104A V105A) (spec)*	This study	Fig. 5
BYB1230	Δ*lytE::lox72, yvbJ::PxylA-lytE (cat) sweDC::kan, ycgO::Pspank-sweDC(ΔLysM) (spec)*	This study	Fig. 5
BYB1231	*sweDC::kan, ycgO::Pspank-sweDC (erm)*	This study	Fig. 5
BYB1232	*sweDC::kan, ycgO::Pspank-sweDC(Δ25-57) (spec)*	This study	Fig. 5
BYB1234	*sweDC::kan, ycgO::Pspank-sweDC(Δ74-108) (spec)*	This study	Fig. 5
BYB1235	*sweDC::kan, ycgO::Pspank-sweDC(D104A V105A) (spec)*	This study	Fig. 5
BYB1236	*sweDC::kan, ycgO::Pspank-sweDC(ΔLysM) (spec)*	This study	Fig. 5
BYB1446	*sacA::Pveg-mCherry (tet), lytE::cat, yvbJ::Pspank-lytE (spec), sweDC::erm*	This study	Fig. S3
BYB1447	*sacA::Pveg-mCherry (tet), lytE::cat, yvbJ::Pspank-lytE (spec), cwlO::kan, sweDC::erm*	This study	Fig. S3
BYB1448	*walH::tet*	This study	
