# Supplementary material for: SweC and SweD are essential co-factors of the FtsEX-CwlO cell wall hydrolase complex in Bacillus subtilis
Source: PLoS Genet. 2019 Aug 22;15(8):e1008296. doi: 10.1371/journal.pgen.1008296 (PMC6705773; doi:10.1371/journal.pgen.1008296)
Supplement: S2 Table — All plasmids and their sources are listed in this table. (PDF) [file pgen.1008296.s014.pdf]

TABLE S2: Plasmids used in this study

Plasmid	Description	Source
pYB02	ycgO::Pspank-sweDC (erm)	This study
pYB04	His6-SUMO-sweC-soluble domain (amp)	This study
pYB05	His6-SUMO-sweD-soluble domain (amp)	This study
pYB16	amyE::sweD-sfGFP (kan)	This study
pYB20	ycgO::Pspank-sweD (spec)	This study
pYB21	ycgO::Pspank-sweC (spec)	This study
pYB61	yvbJ::PxylA-lytE (cat)	This study
pYB130	ycgO::Pspank-sweDC(Δ25-57) (spec)	This study
pYB132	ycgO::Pspank-sweDC(Δ74-108) (spec)	This study
pYB134	ycgO::Pspank-sweDC(D104A V105A) (spec)	This study
pYB135	ycgO::Pspank-sweDC(ΔLysM) (spec)	This study
pDR244	Ppa-cre, ori(ts) (spec) (amp)	Wang *et al*. (2014)
pWX470	lox66-kan-lox71 (amp)	Meeske *et al.* (2016)
pER65	ycgO::Pspank (erm)	Meisner et al. (2013)
pTD68	His6-SUMO (amp)	Morlot *et al.* (2010)
pWX467	lox66-erm-lox71 (amp)	Wang *et al*. (2014)
pYB24	T25-FtsX (kan)	This study
pYB26	T18-FtsX (amp)	This study
pYB29	FtsE-T25 (kan)	This study
pYB30	T18-FtsE (amp)	This study
pYB42	T25-FtsEX (kan)	This study
pYB44	T18-FtsEX (amp)	This study
pYB36	sweD-T25 (kan)	This study
pYB37	sweD-T18 (amp)	This study
pYB39	sweC-T18 (amp)	This study
pYB147	sweD(Δ25-57)-T18 (amp)	This study
pYB183	sweD(TM)-T18 (amp)	This study
pEB352	T25-TolB (kan)	Battesti & Bouveret (2008)
pEB356	T18-Pal (amp)	Battesti & Bouveret (2008)
pEB354	pUT25 (kan)	Gully & Bouveret (2006)
pEB355	pUT18 (amp)	Gully & Bouveret (2006)
pWX469	lox66-tet-lox71 (amp)	Meeske *et al.* (2016)
