# Supplementary material for: SweC and SweD are essential co-factors of the FtsEX-CwlO cell wall hydrolase complex in Bacillus subtilis
Source: PLoS Genet. 2019 Aug 22;15(8):e1008296. doi: 10.1371/journal.pgen.1008296 (PMC6705773; doi:10.1371/journal.pgen.1008296)
Supplement: S3 Table — All oligonucleotides used for plasmid construction, gene deletion, or sequencing are listed in this table. Capital letters were used for restriction endonuclease recognition sites and underlined letters indicate mutated bases. (PDF) [file pgen.1008296.s015.pdf]

TABLE S3: Oligonucleotide primers used in this study

Primers	Sequence
oYB33	CGCACTAGTTTATCGTGTTAACGTTTTTACGATC
oYB34	GCTCTGGCTTTTTTCTTACACC
oYB35	CTGAGCGAGGGAGCAGAAGGAGTTTCATCACCTTCTATTCTTTTTTTATCC
oYB36	GTTGACCAGTGCTCCCTGGTCAAAAACAGGCTGACTCAGCCTG
oYB37	CGATTCCTTCTATCATCACAGG
oYB38	GTGAAGCTTAGAAGGTGATGAAACATGGTGGAAATTGCTATTATTGCGTTG
oYB41	GCGGGATCCCTGACTGACGAAGACCAGGCC
oYB42	CGCCTCGAGTTATCGTGTTAACGTTTTTACGATCTTTTTG
oYB43	GCGGGATCCGACCCGATGAAAGATGTAGAACAGG
oYB44	CGCCTCGAGTCATACGAGCACCTTCTCATTATCTTTG
oYB61	GTAGCGACCGGCGCTCAGGATCCGATTATGTCGAATGTGTCCCTAAAAAAG
oYB62	AGATCCGGAACCTGATCCTGAAGGTCCTACGAGCACCTTCTCATTATCTTTG
oYB63	GGACCTTCAGGATCAGGTTCCGGATCTGGCAGTAAAGGTGAAGAACTGTTCACC
oYB64	CCAGGGGTACCGAGCTCGAATTCTTATTTGTAGAGTTCATCCATGCCG
oYB67	CGCACTAGTTCATACGAGCACCTTCTCATTATC
oYB68	GTGAAGCTTAGAAGGTGATGAAACATGACAAAACGGGGCATTC
oYB83	TTCAAGCTTCTTCCGAAACTTACTG
oYB84	CTGAGCGAGGGAGCAGAAGGACAAATGAAAAGCCGTCCCGAAAAC
oYB85	GTTGACCAGTGCTCCCTGGTCCCTTCTTTTTCTTCTATGATTACATGAAATCTC
oYB86	GAAATAGCCATCCTCACGTCC
oYB91	CTGCAGGGTCGACTCTAGAATTCATGATTAAAATTCTCGGGCGCC
oYB92	CACTGCAGGTCGACTCTAGAATTCATGATTAAAATTCTCGGGCGCC
oYB93	GTCGACGGTATCGATAAGCTTGATATCTTATACTCGCAGAAACTTGCGG
oYB95	CTGGCGGCTAAGCTTGGCGTAATTACTCGCAGAAACTTGCGGATG
oYB97	CTGCAGGGTCGACTCTAGAATTCATGATAGAGATGAAGGAAGTATATAAAGCC
oYB98	CACTGCAGGTCGACTCTAGAATTCATGATAGAGATGAAGGAAGTATATAAAGCC
oYB99	GTCGACGGTATCGATAAGCTTGATATCTTAATCATATGAACCATACTCCCC
oYB100	GTAACCAGCCTGATGCGATTGCTGATCATATGAACCATACTCCCCTCTTG
oYB102	CACACAGGAAACAGCTATGACCATGATAGAGATGAAGGAAGTATATAAAGCCTATC
oYB109	GTAACCAGCCTGATGCGATTGCTGTACGAGCACCTTCTCATTATCTTTG
oYB110	CTGGCGGCTAAGCTTGGCGTAATTACGAGCACCTTCTCATTATCTTTG
oYB111	CACACAGGAAACAGCTATGACCATGGTGGAAATTGCTATTATTGCGTTG
oYB113	CTGGCGGCTAAGCTTGGCGTAATTCGTGTTAACGTTTTTACGATC
oYB114	CACACAGGAAACAGCTATGACCATGATGACAAAACGGGGCATTCAG
oYB127	CAGCACACGCGAGCTCTTG
oYB128	CTGAGCGAGGGAGCAGAAGGATTTTCACGCTTCATCCCAATCATC
oYB129	GTTGACCAGTGCTCCCTGGTCACAGTGGAGTGGAATAAGACAAAATC
oYB130	CATTAATACGGCACCGACAATCAC
oYB134	CTGGCGGCTAAGCTTGGCGTAATCTGTTCTACATCTTTCATCGGGTCTC
oYB141	CTGAGCGAGGGAGCAGAAGGAATAATAGCAATTTCCACGTTTCATCACC
oYB142	GTTGACCAGTGCTCCCTGGTCATCAAAGATAATGAGAAGGTGCTCG
oYB143	CTGAGCGAGGGAGCAGAAGGATTGTCATACGAGCACCTTCTCATTATC
oYB162	ATGAAATAAAATGCATCTGTATTTGAATGAAGCTTGTTAACATTTGGGGAGGAAAATATG
oYB163	AAGGGTAACTATTGCCGTATGGGATCCATCTCGAGCAATGAACGGGTTTTCTCTAAAAATTAG
oYB313	GTGAGCGGATAACAATTAAGCTTAGAAGGTGATGAAACATGGTGGAAATTGC
oYB314	GAGCTAGCATCTGCAGTTACTAGTTTATCGTGTTAACGTTTTTACGATCTTTTTG
oYB315	CAAGGTTTGTTTCCAGGCCTGATCCCGGGTCTCTTTGAGAATATGAGAATGC
oYB316	TTCTCAAAGAGACCCGGGATCAGGCCTGGAAACAAACCTTGTCATTCGC
oYB319	CTTCTCATTATCGCCTGATCCATTGATGTCGTTCTGCTTTGATTTG
oYB320	GAACGACATCAATGGATCAGGCGATAATGAGAAGGTGCTCGTATGAC
oYB324	GTATCAGTGGAAGCAGCAAATACGATTATCAAAGATAATGAGAAGGTGC
oYB325	CGAGCTAGCATCTGCAGTTACTAGTTTATCGTGTGACTTTGTCTGATTTCGTGTCTGAG
oYB370	GATCCGGATACGTCCTGGGAAGTCATGAAGACGCTGGAGGAAATTAAC
oYB371	GTTAATTTCCTCCAGCGTCTTCATGACTTCCCAGGACGTATCCGGATC

oYB372	GAAACACATGGATGACATTTGCATCCATTAGTGCTGTAACCGTTACGCTG
oYB373	CAGCGTAACGGTTACAGCACTAATGGATGCAAATGTCATCCATGTGTTTC
oJM28	TTCTGCTCCCTCGCTCAG
oJM29	CAGGGAGCACTGGTCAAC
oJM54	TGCTATCGGAGAGCATTGG
oJM103	ATTAAGCTTGTCCCGGGTAAGTTAACATTTGGGGAGGAAAATATG
oJM104	CCACCGAATTAGCTTGCATGCAATGAACGGGTTTTCTCTAAAAATTAG
